# Supplementary material for: On the patterns of genetic intra-tumor heterogeneity before and after treatment
Source: Genetics. 2025 May 29;230(4):iyaf101. doi: 10.1093/genetics/iyaf101 (PMC12341898; doi:10.1093/genetics/iyaf101)
Supplement: iyaf101_Supplementary_Data [file iyaf101_supplementary_data.pdf]

Supplemental material for 'On the patterns of genetic intra-tumour heterogeneity before and after treatment'

Alexander Stein and Benjamin Werner

This PDF file includes

- Supporting text
- Supplementary Figs. S1 to S7
- Supplementary Tables S1 to S5

Supporting text

A Probability mass functions of the birth-death process

Given constant rates for birth b and death d, and starting with a cells, what is the probability $p(a \to n, t)$ that we have n cells after time t? – A comprehensive answer to this question is given by Bailey (1991). Here, we summarize the results with addition of more recent results that circumvent issues with computational stability and efficiency.

A.1 Exact solutions

For general birth and death rates, $b \geq 0$ and $d \geq 0$, and general initial population size $a \geq 1$, the probability to grow to size n in time t is given by

$$\begin{aligned} p(a \to n, t) &= \sum_{j=0}^{\min(a,n)} \binom{a}{j} \binom{a+n-j-1}{a-1} \alpha^{a-j} \beta^{n-j} (1-\alpha-\beta)^j \quad \text{for } n \geq 1 \\ p(a \to 0, t) &= \alpha^a. \end{aligned} \tag{A1}$$

In the super- and subcritical case $b \neq d$, we have

$$\alpha = \frac{de^{rt} - d}{be^{rt} - d} \quad \text{and} \quad \beta = \frac{be^{rt} - b}{be^{rt} - d}, \tag{A2}$$

and in the critical case $b = d$, we have

$$\alpha = \beta = \frac{bt}{1+bt}. \tag{A3}$$

The mean and variance is given by

$$\begin{aligned} m(t) &= ae^{(b-d)t} \\ \sigma^2(t) &= a\frac{b+d}{b-d} e^{(b-d)t} (e^{(b-d)t} - 1) \end{aligned} \tag{A4}$$

Whereas it is remarkable that an exact solution can be obtained, the solution is computationally expensive and unstable. If the term $(1-\alpha-\beta)$ is negative and the binomial coefficients are large, the limits of machine precision is exceeded and one yields wrong values for $p(a \to n, t)$. An alternative form has been derived that sums over positive terms only (Tavaré 2018) that reads

$$p(a \to n, t) = \sum_{j=0}^{\min(a,n)} \binom{a}{j} (1-\alpha)^j \alpha^{a-j} p^*_{jm} \tag{A5}$$

with

$$p^*_{jm} = \binom{n-1}{n-j} (1-\beta)^j \beta^{(n-j)} \tag{A6}$$

for $j \geq 0$ and $p^*_{00} = 1$ and $p^*_{0m} = 0$ for $j = 0$. Whereas the sum in eqn. (A5) is computationally stable, it is still computationally expensive for large values of a or n.

If the initial population size is $a = 1$ and $b \neq d$, the probability to grow to size n after time t simplifies to

$$\begin{aligned} p(1 \to n, t) &= (1-\alpha)(1-\beta)\beta^{n-1} \quad \text{for } n \geq 1 \\ p(1 \to 0, t) &= \alpha \end{aligned} \tag{A7}$$

with α and β given by eqn. (A2). The distribution is recognized as generalized geometric distribution.

For the critical case ($b = d$) with initial population size $a = 1$, the probability mass becomes

$$\begin{aligned} p(1 \to n, t) &= \frac{(bt)^{n-1}}{(1+bt)^{n+1}} \quad \text{for } n \geq 1 \\ p(1 \to 0, t) &= \frac{bt}{1+bt}. \end{aligned} \tag{A8}$$

For the pure-birth process ($d = 0$) and general initial population size $a \geq 1$, we have

$$p(a \to n, t) = \binom{n-1}{a-1} e^{-abt}(1-e^{-bt})^{n-a} \tag{A9}$$

which is a negative binomial distribution, i.e. the probability of having n trials given a successes with success probability $p = e^{-bt}$.

For the pure death process $b = 0$, we have

$$p(a \to n, t) = \binom{a}{n} e^{-ndt}(1-e^{dt})^{a-n}, \tag{A10}$$

which is a binomial distribution, i.e. the probability of having n successes in a Bernoulli trials with success probability $p = e^{-dt}$.

A.2 Saddlepoint approximation method

The saddlepoint approximation was introduced to approximate the probability density function or probability mass function given that we know the moment generating function and has found many applications (Daniels 1954; Butler 2007).

Consider a discrete random variable X that can take values of integers, $k = 1, 2, 3, \ldots$. Let us denote the moment generating function by $M(\theta)$, and the cumulant generating function $K(\theta) = \log(M(\theta))$. Then the saddlepoint mass function is computed by

$$\hat{p}(k) = \frac{1}{\sqrt{2\pi K''(\hat{\theta})}} e^{K(\hat{\theta}) - \hat{\theta}k} \tag{A11}$$

where $\hat{\theta}$ is the solution to $K'(\hat{\theta}) = k$. Noteworthy, by design the saddlepoint approximation is not normalized. Once normalized, one talks about the normalized saddlepoint mass function.

Davison *et al.* (2021) applied the saddlepoint approximation on the known moment generating function given by Bailey (1991). For the super- and subcritical case ($b \neq d$) the saddlepoint mass function is given

by

$$\hat{p}(a \to n,t) = \frac{1}{\sqrt{2\pi a}} \frac{1}{\tilde{s}^k} \left(\frac{d - b\tilde{s} + d(\tilde{s}-1)e^{(b-d)t}}{d - b\tilde{s} + b(\tilde{s}-1)e^{(b-d)t}} \right) \left(- \frac{(e^{(b-d)t}-1)e^{(b-d)t}\tilde{s}(b-d)^2 \left(-b^2\tilde{s}^2 + be^{(b-d)t}d(\tilde{s}^2-1)+d^2\right)}{\left(b(e^{(b-d)t}(\tilde{s}-1)-\tilde{s})+d\right)^2 \left(b\tilde{s}+d(-e^{(b-d)t}\tilde{s}+e^{(b-d)t}-1)\right)^2} \right)^{-1/2} \tag{A12}$$

where

$$\tilde{s} = \frac{1}{2A}(-B+\sqrt{B^2-4AC}) \quad \text{with} \begin{cases} A = b(e^{(b-d)t}-1)(b-de^{(b-d)t}) \\ B = 2bd(1+e^{2(b-d)t}-e^{(b-d)t}-\frac{a}{n}e^{(b-d)t}) + e^{(b-d)t}(b^2+d^2)(\frac{a}{n}-1) \\ C = d(e^{(b-d)t}-1)(d-be^{(b-d)t}) \end{cases} \tag{A13}$$

For the critical case $(b = d)$, it is

$$\hat{p}(a \to n,t) = \frac{1}{\sqrt{2\pi a}} \frac{1}{\tilde{s}^k} \left(\frac{bt(1-\tilde{s})+\tilde{s}}{1-bt(\tilde{s}-1)} \right)^a \times \left(\frac{bt\tilde{s}\left(-bt\tilde{s}^2+bt+\tilde{s}^2+1\right)}{\left(bt(\tilde{s}-1)-1\right)^2\left(-bt\tilde{s}+bt+\tilde{s}\right)^2} \right)^{-1/2} \tag{A14}$$

where

$$\tilde{s} = \frac{1}{2A}(-B+\sqrt{B^2-4AC}) \quad \text{with} \begin{cases} A = bt-(bt)^2 \\ B = 2(bt)^2+\frac{a}{n}-1 \\ C = -bt-(bt)^2 \end{cases} \tag{A15}$$

Davison *et al.* (2021) have validated the approximation over a range of parameters. They have found that the approximation yields good results for sufficiently large n and a. Furthermore, their analysis shows that normalization of the saddlepoint mass function does not lead to significant improvement of the approximation such that we continue working with the unnormalized probabilities.

A.3 Heuristic approximation using over-dispersed Poisson distributions

We start by observing that for the pure-birth process, the probability mass follows a binomial distribution. In the pure-death process, it follows a negative binomial distribution which is commonly used to describe overdispersed count data. Furthermore, plotting eqn. (A5) for general b and d and sufficiently large n shows the typical bell shape similar to a Poisson distribution but with increased variance. Those observation motivated us to approximate the probability mass with an overdispersed Poisson distribution that we parameterise with the expressions for the mean and variance.

Consul Poisson distribution: We chose the Consul Poisson distribution (Consul and Jain 1973) as one of the simplest formulas of an overdispersed Poisson distribution that reads

$$P(k) = \frac{\mu e^{-k\lambda-\mu}(k\lambda+\mu)^{k-1}}{k!} \tag{A16}$$

and has mean $\frac{\mu}{1-\lambda}$ and variance $\frac{\mu}{(1-\lambda)^3}$. In the case $\lambda = 0$, we have the standard Poisson distribution.

We parameterize the Poisson distribution with the mean and the variance of the super- or subcritical birth-death process given in eqn. (A4) that leads to

$$\begin{aligned}\frac{\mu}{1-\lambda} &= m(t) = ae^{rt} \\ \frac{\mu}{(1-\lambda)^3} &= \sigma^2(t) = a\frac{b+d}{b-d}e^{(b-d)t}(e^{(b-d)t}-1)\end{aligned} \tag{A17}$$

Solving this equation for μ and λ has two solutions. We chose the solution leading to positive parameters μ and λ in case of overdispersion (i.e. $\sigma^2(t) > m(t)$) that is

$$\begin{aligned}\mu &= \frac{m^{3/2}(t)}{\sigma(t)} = \frac{\left(ae^{(b-d)t}\right)^{3/2}}{\sqrt{\frac{a(b+d)e^{(b-d)t}\left(e^{(b-d)t}-1\right)}{b-d}}} \\ \lambda &= \frac{\sigma(t)-m^{1/2}(t)}{\sigma(t)} = \frac{\sqrt{\frac{a(b+d)e^{(b-d)t}\left(e^{(b-d)t}-1\right)}{b-d}}-\sqrt{ae^{(b-d)t}}}{\sqrt{\frac{a(b+d)e^{(b-d)t}\left(e^{(b-d)t}-1\right)}{b-d}}}\end{aligned} \tag{A18}$$

Negative binomial distribution: The negative binomial distribution is a standard choice to describe an overdispersed Poisson distribution. It reads

$$P(k) = \binom{k+r-1}{k}(1-p)^k p^r, \tag{A19}$$

has mean $\frac{r(1-p)}{p}$ and variance $\frac{r(1-p)}{p^2}$. It converges towards a Poisson distribution in the limit $p \to 1$. The parameters p and r can be parameterized and expressed in terms of the mean and variance,

$$\begin{aligned}p &= \frac{m(t)}{\sigma^2(t)} \\ r &= \frac{m^2(t)}{\sigma^2(t)-m(t)},\end{aligned} \tag{A20}$$

where the mean $m(t)$ and variance $\sigma^2(t)$ are again taken from eqn. (A4).

B Probability for the time to reach size N

The probability for the time to reach size N starting from one cell ($a = 1$) was derived by Durrett (2015). The probability density is approximately

$$f_{T_N}(t) = e^{-\frac{b-d}{b}Ne^{-(b-d)t}}\frac{(b-d)^2N}{b}e^{-(b-d)t}. \tag{B1}$$

This is a Gumbel distribution, which is commonly written in terms of a location parameter μ and shape parameter β. We can write the density as

$$f_{T_N}(t) = \frac{1}{\beta}e^{-\left(z+e^{-z}\right)} \quad \text{with} \quad z = \frac{x-\mu}{\beta}, \tag{B2}$$

such that $\beta = \frac{1}{b-d}$ and $\mu = \frac{1}{b-d} \ln\left(\frac{b-d}{b} N\right)$.

We can then make use of the known properties of the Gumbel distribution. In particular, the expectation is

$$E[T_N] = \mu + \beta\gamma = \frac{1}{b-d}\left(\ln\left(\frac{b-d}{b}N\right) + \gamma\right) \tag{B3}$$

where $\gamma \approx 0.577$ is the Euler constant. Further, the moment-generating function defined by

$$\phi(u) = E[e^{uT_N}] = \int_{-\infty}^{\infty} e^{ut} f_{T_N}(t)\, dt \tag{B4}$$

takes the form

$$\phi(u) = \Gamma(1-\beta u)e^{\mu u} = \Gamma\left(1 - \frac{u}{b-d}\right)\left(\frac{b-d}{b}N\right)^{\frac{u}{b-d}}, \tag{B5}$$

where $\Gamma(z) = \int_0^\infty t^{z-1} e^{-t}\, dt$ is the gamma function.

The Gumbel distribution is defined over $(-\infty, \infty)$ whereas we are only interested in positive times $T_N > 0$. However, in applications the integral over the density for negative t is typically negligible and we proceed with approximating $\int_{-\infty}^0 f_{T_N}(t)\, dt = 0$.

C Switching between fixed-time and fixed-size conditioning

The random process is stopped at fixed time $T = t$ or fixed size $N = n$ and an observable is measured. We know that the probability to grow to size n in time t (Supplementary Section A) that we write as $p(n|t)$ to be explicit on the conditioning. Similarly, starting from a single cell, we know the probability for the time t to grow to size n for the first time (Supplementary Section B) that we denote by $f_T(t|N = n)$. We investigate the expectation of an observable O.

Given we know the expected observation for fixed time $T = t$, we can compute the expected observation for fixed size $N = n$ by

$$\begin{aligned} E[O|N = n] &= \int_0^\infty E[E[O|N = n]|T = t]\, f_T(t|N = n)\, dt \\ &= \int_0^\infty E[O|T = t]\, f_T(t|N = n)\, dt. \end{aligned} \tag{C1}$$

In the first equality, we decompose the expectation of $E[O|N = n]$ into expectations for all possible times t. In the second equality, we use the law of total expectation which states $E[E[O|N = n]|T = t] = E[O|T = t]$.

Similarly, if we know the expected observation for fixed size $N = n$, we can compute the expected observation for fixed time $T = t$ by

$$E[O|T = t] = \sum_{n=1}^{\infty} E[O|N = n]\, p(n|t), \tag{C2}$$

where we condition on survival and thus chose $n = 1$ rather than $n = 0$.

D Proof of Theorem 1

Part 1: We prove eqn. (7) for general N_0 following Appendix B of Gunnarsson *et al.* (2021) who proved the result for $N_0 = 1$. Restricted (but not conditioned) on survival of the entire population $\{Z_0(t) > 0\}$, the expected number of mutations that emerged in the time interval $[t', t' + dt']$ and grow to size k in the remaining time $t - t'$ was shown to be

$$E\left[\left(S_k^{(new)}(t'+dt') - S_k^{(new)}(t')\right) 1_{\{Z_0(t)>0\}}\right] = bE[\Omega]E[Z_0(t')]p(1 \to k, t-t')\, dt'. \tag{D1}$$

Here, Ω is the number of mutations generated in a division. Given our *Theoretical framework*, we have $E[\Omega] = 2m$. The expected population size in the birth-death process is given by $E[Z_0(t')] = N(t') = N_0 e^{(b-d)t'}$. Conditioning the total population to survive time t leads to

$$E\left[\left(S_k^{(new)}(t'+dt') - S_k^{(new)}(t)\right) |Z_0(t) > 0\right] = \frac{1}{1-p(N_0 \to 0, t)} 2mbN(t')p(1 \to k, t-t')\, dt'. \tag{D2}$$

It remains to integrate over the time interval of entire time interval $t' \in [0, t]$ such that

$$E[S_k^{(new)}] = \frac{1}{1-p(N_0 \to 0, t)} \int_0^t 2mbN(t')p(1 \to k, t-t')dt', \tag{D3}$$

which makes the proof complete.

Noteworthy, the expected BWD is given by

$$E[W_k^{(new)}] = E[S_k^{(new)}]/m = \frac{1}{1-p(N_0 \to 0, t)} \int_0^t 2bN(t')p(1 \to k, t-t')dt', \tag{D4}$$

Part 2: Next, we prove eqn. (8). We start by writing the initial BWD as $W_k^{(init)}$, label branches of the phylogenetic tree with $i = 1, 2, ...L$ and their branch widths with k_i. During time t, branches grow stochastically and we filter for those that grow to size k by writing

$$W_k^{(pre)} = \sum_{i=1}^{L} 1_{\{k_i \to k\}} = \sum_{k'=1}^{N_0} \sum_{j=1}^{W_{k'}^{(init)}} 1_{\{k_j \to k\}} \tag{D5}$$

where $1_{\{k_i \to k\}} = 1$ if branch i of width k_i grows to size k and $1_{\{k_i \to k\}} = 0$ otherwise. In the second equality, we used that the L branches are partitioned into N_0 classes $\{W_{k'}^{(init)}\}_{k'=1}^{N_0}$ according to their initial width k'.

Taking the expectation, we have

$$\begin{aligned} E[W_k^{(pre)}] &= E\left[\sum_{k'=1}^{N_0} \sum_{j=1}^{W_{k'}^{(init)}} 1_{\{k_j \to k\}}\right] \\ &= \sum_{k'=1}^{N_0} E\left[\sum_{j=1}^{W_{k'}^{(init)}} 1_{\{k_j \to k\}}\right] \\ &= \sum_{k'=1}^{N_0} E[W_{k'}^{(init)}]\, p(k' \to k, t). \end{aligned} \tag{D6}$$

In the second equality, we used linearity of the expectation. In the third equality, we used Wald's identity together with $E[1_{\{k_j \to k\}}] = p(k' \to k, t)$ when k_j has width k'. We substitute $E[W_k^{(pre)}] = E[S_k^{(pre)}]/m$ and $E[W_k^{(init)}] = E[S_k^{(init)}]/m$ and condition the entire population on survival such that

$$E[S_k^{(pre)}] = \frac{1}{1 - p(N_0 \to 0, t)} \sum_{k'=1}^{N_0} E[S_{k'}^{(init)}]\, p(k' \to k, t), \tag{D7}$$

which is the desired result.

E Site frequency spectra at detection

E.1 Fixed-time expectation

Starting from a single cell with u_0 mutations, the population grows time t_d to detection. The average growth in the time interval $[0, t_d]$ is given by $N(t') = e^{(b_1-d_1)t'}$ and the expected population size at detection conditioned on survival is $N_d = \tilde{N}(t_d) = \frac{1}{1-\alpha(t_d)} e^{(b_1-d_1)t_d}$.

Newly emerging mutations: Following Theorem 1, we have

$$E[S_k^{(new)}] = \frac{1}{1-\alpha(t_d)} \int_0^{t_d} 2mb_1 e^{(b_1-d_1)t'} p(1 \to k, t_d - t')\, dt'. \tag{E1}$$

Following Ohtsuki and Innan (2017) and Gunnarsson *et al.* (2021), the integral is rewritten and simplified for the limits $N_d \to \infty$ and $k \to \infty$ giving us

$$\begin{aligned} E[S_k^{(new)}] &= 2mN_d \int_0^{1-1/N_d} \left(1 - \frac{d_1}{b_1} y\right)^{-1} (1-y) y^{k-1}\, dy \\ &\to 2mN_d \sum_{l=0}^{\infty} \frac{\left(\frac{d_1}{b_1}\right)^l}{(k+l)(k+l+1)} \quad \text{for } N_d \to \infty \\ &\to 2mN_d \frac{b_1}{b_1 - d_1} \frac{1}{k(k+1)} \quad \text{for } k \to \infty. \end{aligned} \tag{E2}$$

Considering only mutations with infinite lineage, one can use the probability mass functions of the pure-birth process (Gunnarsson *et al.* 2021), in which case the SFS is

$$E[S_k^{(new)}] = 2m \frac{b_1}{b_1 - d_1} N_d \left(1 - \frac{1}{N_d}\right)^k \left(\frac{1}{k(k+1)} + \frac{1}{N_d} \frac{1}{k+1}\right). \tag{E3}$$

Since all mutations have an infinite lineage in the pure-birth process, this formula also provides the SFS for the pure-birth process when setting $d_1 = 0$.

Preexisting mutations: Following Theorem 1, we have

$$E[S_k^{(pre)}] = \frac{1}{1-\alpha(t_d)} u_0 p(1 \to k, t_d) = u_0 (1 - \beta(t_d)) \beta(t_d)^{k-1}, \tag{E4}$$

which is just the number of initial mutations multiplied by the probability of growing from size 1 to size k in time t_d conditioned on survival. $\beta(t_d)$ is defined in eqn. (A2).

Our main interest is in mutation accumulation during tumour progression. Therefore, we work with $u_0 = 0$ in the main text leading to $E[S_k^{(pre)}] = 0$ and $E[S_k^{(det)}] = E[S_k^{(new)}]$.

E.2 Fixed-size expectation

Following eqn. (C1), the expected SFS at fixed size $N = N'_d$ can be expressed in terms of the expected SFS at fixed time $T = t$ by writing

$$\begin{aligned} E[S_k|N = N'_d] &= \int_0^\infty E[S_k|T = t]\, f_T(t|N = N'_d)\, dt \\ &\approx \int_0^\infty C\tilde{N}(t)\, f_T(t|N = N'_d)\, dt. \end{aligned} \tag{E5}$$

In the second line, we used the solution for large population size ($\tilde{N}(t) \to \infty$ in eqn. (9)) such that $E[S_k|T = t]$ is proportional to the expected size at time t. Here, C is independent of time and reads

$$C = 2m \sum_{l=0}^{\infty} \frac{\left(\frac{d_1}{b_1}\right)^l}{(k+l)(k+l+1)}. \tag{E6}$$

The expected size at fixed time t_d can be written as

$$N_d = \tilde{N}(t_d) = \frac{1}{\sigma} e^{(b_1-d_1)t} - \frac{\rho}{\sigma} \tag{E7}$$

with $\rho = \frac{d_1}{b_1}$ and $\sigma = \frac{b_1-d_1}{b_1}$ (eqn. 17 in (Gunnarsson *et al.* 2021)). The integral can be expressed in terms of the moment generating function (eqn. (B5)) such that

$$E[S_k|N = n] = C\left(\frac{1}{\sigma}\phi(b_1 - d_1) - \frac{\rho}{\sigma}\right). \tag{E8}$$

However, we have

$$\phi(b_1 - d_1) = \Gamma\left(1 - \frac{b_1 - d_1}{b_1 - d_1}\right)\left(\frac{b_1 - d_1}{b_1} N'_d\right)^{\frac{b_1-d_1}{b_1-d_1}} \tag{E9}$$

and $\Gamma(0)$ is not defined.

To avoid the convergence problem, we consider the normalized SFS that we define by $E[\tilde{S}_k|T = t] = \frac{E[S_k|T=t]}{E[Z_0(t)|Z_0(t)>0]} = C$. Again, we switch from the fixed-time expectation to the fixed-size expectation, which yields

$$E[\tilde{S}_k|N = N'_d] = \int_0^\infty C f_T(t|N = N'_d)\, dt = C = E[\tilde{S}_k|T = t]. \tag{E10}$$

We find that the fixed-time expectation and fixed-size solution expectation. The perfect one-to-one correspondence vanishes if we take the exact expression for the fixed-time expectation instead of the large-size approximation in eqn. (9).

F Site frequency spectrum after treatment with homogeneous response

We compute the expected SFS after treatment with homogeneous treatment response. As initial condition, we consider fixed size N_d' and the expected SFS at detection. For the initlal SFS, we approximate the fixed-size expectation of the SFS with the fixed-time expectation of the SFS by replacing the expected size $N_d = \tilde{N}(t_d)$ with the fixed-size N_d' in eqn. (9). We denote the treatment time by t_f after which the population conditioned on survival has expected size $N_f = \frac{1}{1-p(N_d' \to 0)} N_d' e^{(b_2-d_2)t_f}$.

F.1 Decreasing cell populations

We consider decreasing cell populations characterized by $b_2 < d_2$ and $N_f < N_d'$.

Newly emerging mutations: Following Theorem 1, we have

$$E[S_k^{(new)}] = \frac{1}{1-p(N_d' \to 0, t_f)} \int_0^{t_f} 2mb_2 N_d' e^{(b_2-d_2)t'} p(1 \to k, t-t')\, dt'. \tag{F1}$$

Here, $p(1 \to k, t-t')$ is given by eqn. (A7). Using the same transformation as for the exponential growth starting from a single cell (eqn. (B.5) to (B.6) in (Gunnarsson *et al.* 2021)), the integral can be rewritten as

$$E[S_k^{(new)}] = 2mN_f \int_0^{\frac{e^{(b_2-d_2)t_f}-1}{e^{(b_2-d_2)t_f}-\frac{d_2}{b_2}}} \left(1 - \frac{d_2}{b_2} y\right)^{-1} (1-y) y^{k-1}\, dy. \tag{F2}$$

In general, the upper integral bound does not go to 1 such that we cannot transform the integral into a sum as done in ref. Gunnarsson *et al.* (2021). Instead, we solved the integral numerically.

The situation changes in the limit $b_2 \to d_2$. Then, the upper integral bound becomes 1 but at the same time the integrand becomes y^{k-1} resulting in $E[S_k^{(new)}] = 2mN_f \frac{1}{k}$. This result coincides with the equilibrium solution for $b_2 = d_2$ that will be further refined in the next subsection (see eqn. (F5)).

In the limit $\frac{d_2}{b_2} \to \infty$, the upper integral bound converges to 0 whereas the integrand converges to $\frac{1}{1-\infty y}(1-y)y^{k-1}$ resulting in $E[S_k^{(new)}] = 0$. The result is intuitive. If there are no birth events, then there are no new mutations.

Preexisting mutations: Following Theorem 1, we have

$$E[S_k^{(pre)}] = \frac{1}{1-p(N_d' \to 0, t_f)} \sum_{k'=1}^{N_d'} E[S_{k'}^{(det)}] p(k' \to k, t_f). \tag{F3}$$

Here, $p(k' \to k, t_f)$ is given by eqn. (A1) parameterized with b_2 and d_2. By construction, the initial SFS is the SFS at detection whose expectation is given by eqn. (9).

In general, the exact probabilities $p(k' \to k, t_f)$ take a complicated form. Consequently, the evaluation of $E[S_k^{(pre)}]$ for specific k consists of computing a sum ranging from 0 to N_d' over sums ranging from

0 to $\min(k,k')$. The computational cost is high but can be decreased with the approximation methods discussed in Supplementary Section A.

In the pure-death process with $b_1 = 0$, we can use (A10) that resolves the second sum without the use of approximation methods. Resolving the first sum remains complicated but is possible when we condition on fixed size instead of fixed time (see Supplementary Section G).

F.2 Constant cell populations

Next, we consider cell populations that remain approximately constant characterized by $b_2 = d_2$ and $N_d' \approx N_f$.

Newly emerging mutations: Following Theorem 1, we have

$$E[S_k^{(new)}] = \frac{1}{1-p(N_d'\to 0,t_f)}\int_0^{t_f} b_2 2mN_d' p(1\to k,t_f)\,dt', \tag{F4}$$

where $p(1\to k,t_f)$ are given by eqn. (A8). We solve the integral and obtain

$$E[S_k^{(new)}] = \frac{1}{1-p(N_d'\to 0,t_f)} 2mb_2N_d' \int_0^{t_f} \frac{(b_2t_f)^{k-1}}{(1+b_2t_f)^{k+1}}\,dt' = 2mN_f\left(\frac{b_2t_f}{1+b_2t_f}\right)^k \frac{1}{k}. \tag{F5}$$

As $t\to\infty$, the SFS is proportional to $\frac{1}{k}$, which is known as the equilibrium solution from other constant population size models, namely the Moran process or the Wright-Fisher process Durrett (2008).

Preexisting mutations: Following Theorem 1, we have

$$E[S_k^{(pre)}] = \frac{1}{1-p(N_d'\to 0,t_f)}\sum_{k'=1}^{N_d'} E[S_k^{(det)}]p(k'\to k,t_f). \tag{F6}$$

Here, $p(k'\to k,t_f)$ is given by eqn. (A1) parameterized with b_2. Again, $E[S_k^{(det)}]$ is given by eqn. (9).

Similar to the decreasing population, the computation of $E[S_k^{(pre)}]$ for one specific k consists of evaluating the sum of sums where the second sum can be resolved using the approximations described in Supplementary Section A.

F.3 Increasing cell populations

Eventually, we consider increasing cell populations characterized by $b_2 > d_2$ and $N_f > N_d'$.

Newly emerging mutations: The exact expressions for increasing populations coincide with the ones for decreasing populations such that

$$\begin{aligned}E[S_k^{(new)}] &= \frac{1}{1-p(N_d' \to 0, t_f)} \int_0^{t_f} b_2 2mN_d' e^{(b_2-d_2)t'} p(1 \to k, t-t')\, dt', \\ &= 2mN_f \int_0^{\frac{e^{(b_2-d_2)t_f}-1}{e^{(b_2-d_2)t_f}-\frac{d_2}{b_2}}} \left(1-\frac{d_2}{b_2}y\right)^{-1} (1-y)y^{k-1}\, dy.\end{aligned} \tag{F7}$$

We solved the integral numerically.

In the limit $t_f \to \infty$, the upper integral bound converges to 1 and following Gunnarsson *et al.* (2021) (computations after eqn. (B.6)), we can express the integral as infinite sum

$$E[S_k^{(new)}] = 2mN_f \sum_{l=0}^{\infty} \frac{\left(\frac{d_2}{b_2}\right)^l}{(k+l)(k+l+1)}. \tag{F8}$$

This limit has restricted application. After detection, the cancer is unlikely to continue growing over a long time since the cancer likely becomes lethal by then. However, this scenario is applicable in case birth and death rates are changed before detection, e.g. through activation of the immune system, angiogenesis or other environmental factors.

In the limit $b_2 \to d_2$, we recover the $E[S_k^{(pre)}] = 2mN_f \frac{1}{k}$ like we did for decreasing populations.

In the limit $d_2 \to 0$, we have

$$E[S_k^{(new)}] = 2mN_f \left(1-e^{-b_2 t_f}\right)^k \left(\frac{1}{k(k+1)} + e^{-b_2 t_f} \frac{1}{k+1}\right). \tag{F9}$$

Preexisting mutations: Again, the expressions for increasing populations coincide with the ones for decreasing populations such that

$$E[S_k^{(pre)}] = \frac{1}{1-p(N_d' \to 0, t_f)} \sum_{k=1}^{N_d'} E[S_k^{(det)}] p(k' \to k, t_f), \tag{F10}$$

where $p(k' \to k, t_f)$ is given by eqn. (A1) parameterized with b_2 and d_2, and $E[S_k^{(det)}]$ is given by eqn. (9). Obtaining a single value comes with evaluation of two sums where the second sum can be resolved using approximation methods for $p(k' \to k, t_f)$ in Supplementary Section A.

F.4 Approximating the fixed-size expectation with the fixed-time expectation

For increasing or decreasing populations, we approximate the expected SFS at fixed size N_f' with the expected SFS at fixed time t_f. Therefore, we transform the treatment time into size after treatment using the expression for the expected size after treatment for time t_f giving us

$$N_f' = \frac{1}{1-p(N_d' \to 0, t_f)} N_d' e^{(b_2-d_2)t_f}. \tag{F11}$$

Inverting the exact expression analytically is complicated. However, in applications we have $N'_d > 10^9$ and given that we still have an observable cancer $N_f > 10^3$. For population sizes of this magnitude, we have $p(N'_d \to 0, t_f) \approx 0$ such that $N'_f \approx N'_d e^{(b_2-d_2)t_f}$ and

$$t_f \approx \frac{\log\left(\frac{N_f}{N'_d}\right)}{b_2 - d_2}. \tag{F12}$$

We inserted this expression into the fixed-time expectation to approximate the fixed-size expectation.

G The site frequency spectrum retains its k^{-2} tail after treatment with homogeneous response

After treatment of cancers with homogeneous treatment response, the expected SFS retains its power law tail $E[S_k] \sim k^{-2}$. We assume that mutations that occur in less than 1% of the population are not observable due to sequencing limitations. Thus, we define the tail distribution by S_k with $k \geq k_{\min} = 1\%N$. First, we show that the k^{-2} tail in retained in simulated data that have population sizes in the magnitudes $10^4 - 10^5$. Second, we make a parameter estimation for realistically large tumors and argue why the k^{-2} tail in retained. We show that newly emerging mutations remain at low unobservable frequencies, and observable preexisting mutations are usually large and behave nearly deterministic.

G.1 Observation of the k^{-2} tail in simulated data

We observe a power law of the form $E[S_k] \sim k^{-\gamma}$ from Fig. 2d-f by noting of the plots build a straight line in the loglog-plot for sufficiently large k. Next, we infer the exponent γ from the simulated data using the maximum likelihood estimator from Clauset *et al.* (2009) (see *Material and Methods* in the main text). We first infer exponents of simulated data at detection (Supplementary Fig. S1). As expected, we obtain exponents $\gamma \approx 2$. Depending on the cutoff $k_{\min}$, we observe a small bias towards larger exponents (Supplementary Fig. S1 b, d). This bias may be explained by the high variability in the largest site frequencies. We also note that strictly speaking, we did not show that the SFS of a single realization follows a power law distribution but the expected SFS does.

Next, we inferred the exponents in simulated data after treatment with homogeneous response. Again, we find exponents $\gamma \approx 2$ with small biases towards larger exponents consistent with the inference artefact that we also obtained for the SFS at detection. We conclude the observation of the $S_k \sim k^{-2}$ tail after treatment with homogeneous response.

G.2 Estimating biologically reasonable parameters

We assume that a cancer is detected at population sizes in the range $N_d = 10^9 - 10^{12}$. Assuming a typical division time $\tau_2 = 5\,\text{days}$, the birth rate can be estimated to be $b_1 = \frac{\ln(2)}{\tau_2} \approx 0.14\,\text{days}^{-1}$. For cancers growing to detection size in around 5-15 years, the growth rate is in the range from $0.005 - 0.015\,\text{days}^{-1}$ translating into death rates close to the birth rate in the range $d_2 = 0.125 - 0.135\,\text{days}^{-1}$. In the following, we work with an intermediate death rate $d_1 = 0.13\,\text{days}^{-1}$.

We set treatment time to $t_f = 180$ days. We assume that treatment affects the death rate only such that we keep the birth rate $b_2 = 0.14\,\text{days}^{-1}$. The death rate is adapted according to the three treatment scenarios. In an effective treatment, the cancer may decrease to 1% of its original size translating into death rate $d_2 \approx 0.166\,\text{days}^{-1}$. For approximately constant disease, we have $d_2 = 0.14\,\text{days}^{-1}$. For ineffective treatment, we consider the case in which treatment has no effect at all such that $d_2 = 0.13\,\text{days}^{-1}$.

G.3 Decreasing cell populations

In the pure-death process, the expected SFS has a k^{-2} tail. If $b_2 = 0.0$, then there are no newly emerging mutations such that $E[S_k^{(tot)}] = E[S_k^{(pre)}]$. Instead of conditioning the expectation on fixed time t_f, let us condition on fixed size N'_f starting from fixed detection size N'_d. In the pure-death process, growing from size k' to size k during treatment is the same as sampling k cells from k' cells without replacement. Thus, the probability to grow to size k is given by the hypergeometric distribution,

$$p(k' \to k, N'_d \to N'_f) = \frac{\binom{k'}{k}\binom{N'_f - k'}{N'_d - k}}{\binom{N'_f}{N'_d}}. \tag{G1}$$

If $N'_d \gg N'_f$, the hypergeometric distribution can be approximated by the binomial distribution (sampling with replacement), which is

$$p(k' \to k, N'_d \to N'_f) \approx \binom{N'_f}{k}\left(\frac{k'}{N'_d}\right)^k\left(1 - \frac{k'}{N'_d}\right)^{N'_f - k}. \tag{G2}$$

Interestingly, we arrive at a binomial distribution like the probability mass function for fixed-time in eqn. (A10), although the binomial distribution is different parameterized.

From here, we can follow the derivation of Durrett who studied the form of the sampled SFS to describe frequencies in a sample (Theorem 3 from Durrett (2015)). We transform the sum for the SFS into an integral and focus on the tail of the SFS at detection such that

$$\begin{aligned} E[S_k^{(tot)}] &= \sum_{k'=1}^{N'_d} E[S_{k'}^{(det)}]p(k' \to k, N'_d \to N'_f) \\ &\approx \int_1^{N'_d} \frac{2mN'_d}{k'^2}\binom{N'_f}{k}\left(\frac{k'}{N'_d}\right)^k\left(1 - \frac{k'}{N'_d}\right)^{N'_f - k} dk'. \end{aligned} \tag{G3}$$

We substitute the initial mutant size with its frequency, $x = \frac{k'}{N'_d}$ and $dx = \frac{1}{N'_d}\,dk'$. Furthermore, we assume $N'_d \to \infty$ such that the lower integral boundary goes to 0. We have

$$E[S_k^{(tot)}] = \int_0^1 \frac{2m}{x^2 N'_d}\binom{N'_f}{k} x^k (1-x)^{N'_f - k} (N'_d\,dx). \tag{G4}$$

Using that $\int_0^1 x^a (1-x)^b\,dx = \frac{a!b!}{(a+b+1)!}$ (see Lemma 2 from Durrett (2015)), we obtain

$$E[S_k^{(tot)}] = 2mN'_f \frac{1}{k(k-1)} \quad \text{for} \quad 2 \geq k \geq N'_f, \tag{G5}$$

which scales as k^{-2} for large k. Noteworthy, the continuity approximation works only for sufficiently large k.

The SFS of preexisting mutations retains its k^{-2} tail. Consider a cancer cell population with detection size $N_d = 10^{12}$ that after treatment for time $t_f = 180$ days shrinks to 1% of its original size, $N_f = 10^{10}$. We compute the mean and the standard deviation for a single site frequency that is initially in 1% of the cells, i.e. $k' = 10^{10}$. Inserting the birth and death rates from our parameter estimation $b_2 = 0.14\,\text{days}^{-1}$ and $d_2 = 0.166\,\text{days}^{-1}$ into eqn. (A4), we have

$$\begin{aligned} m(t_f) &= k' e^{(b_2-d_2)t_f} = 10^8 \\ \sigma(t_f) &= \sqrt{k' \frac{b_2+d_2}{b_2-d_2} e^{(b_2-d_2)t_f} (e^{(b_2-d_2)t_f} - 1)} \approx 3.4 \times 10^4 \end{aligned} \tag{G6}$$

Starting from a large populations, we know that the probability mass function takes a bell-shaped curve as long as we are not too close to the extinction boundary (Davison *et al.* 2021). Observing that the standard deviation is multiple magnitudes lower than the mean, we conclude a sharply peaked probability mass function that we interpret as approximately deterministic behaviour.

If the growth dynamics of site frequencies is deterministic, it is straightforward to show that the initial shape of the SFS is retained. Focusing on the tail of the SFS at detection and assuming deterministic growth during treatment, we have

$$E[S_k^{(pre)}] = \sum_{k'=1}^{N_d'} 2mN_d \frac{d_1}{b_1-d_1} \frac{1}{k'^2} \delta\left(k - k' e^{(b_2-d_2)t_f}\right) = 2m \frac{d_1}{b_1-d_2} N_f \frac{1}{k^2}. \tag{G7}$$

Here, we use deterministic growth for the total population $N_f = N_d e^{(b_2-d_2)t_f}$ and approximate the probability mass function by $\delta\left(k - k' e^{(b_2-d_2)t_f}\right)$ that is 1 if $k - k' e^{(b_2-d_2)t_f} = 0$ and 0 otherwise.

Newly emerging mutations are restricted to small frequencies. Since $b_2 < d_2$, newly emerging mutations survive only due to drift. Given $b_2 = 0.14$ and $d_2 \approx 0.166$, the probability to survive time t_f is only $1 - \alpha(t_f) \approx 0.000243$. Further, the probability to reach larger sizes $k > 1$ is decreasing with time.

G.4 Constant cell populations

The SFS of preexisting mutations retains its k^{-2} tail. Consider a cancer cell population with detection size $N_d = 10^{12}$ that remains approximately constant over treatment time $t_f = 180$ days. We compute the mean and the standard deviation for a single site frequency that is initially in 1% of the cells, i.e. $k' = 10^{10}$. Inserting the birth and death rates from our parameter estimation $b_2 = d_2 = 0.14\,\text{days}^{-1}$ into eqn. (A4), we have

$$\begin{aligned} m(t) &= k' = 10^{10} \\ \sigma(t) &= \sqrt{2kb_2t_f} \approx 7.1 \times 10^5 \end{aligned} \tag{G8}$$

With analogous argumentation to the decreasing population, we conclude that the growth is approximately deterministic and the SFS of preexisting mutations retains its k^{-2} tail.

Newly emerging mutations are restricted to small frequencies. Using the same parameters as before, we compute the probability that a newly emerging mutation grows to abundance $k > 100$ during treatment time $t_f = 180$ days

$$1 - \sum_{k'=0}^{100} p(1 \to k', t_f) = 1 - \frac{bt}{1+bt} - \sum_{k'=1}^{100} \frac{(b_2t_f)^{k'-1}}{(1+b_2t_f)^{k'+1}} \approx 1 - 0.9618 - 0.0374 = 0.0008. \tag{G9}$$

The chance is very small, and we conclude that genetic drift alone is not enough to grow to observable site frequencies in the order $k = 10^{10}$.

This is also reflected in the expression $E[S_k^{(new)}] = 2mN_f \left(\frac{b_2 t_f}{b_2 t_f + 1}\right)^k \frac{1}{k}$, which has an exponential cutoff with k. Inserting the parameters above, we note that there is a dominating exponential cutoff with k that reads

$$\left(\frac{b_2 t_f}{b_2 t_f + 1}\right)^k = 0.96^{10^{10}}, \tag{G10}$$

which is extremely small such that $E[S_k^{(new)}]$ is virtually zero for large k.

G.5 Increasing cell populations

The SFS of preexisting mutations retains its k^{-2} tail. Consider a cancer cell population with detection size $N_d = 10^{11}$ that after ineffective treatment for time $t_f = 180$ days continues increasing to approximately $N_f \approx 6 \times 10^{11}$. We compute the mean and the standard deviation for a single site frequency that is initially in 1% of the cells, i.e. $k' = 10^9$. Inserting the birth and death rates from our parameter estimation $b_2 = 0.14\,\text{days}^{-1}$ and $d_2 = 0.13\,\text{days}^{-1}$ into eqn. (A4), we have

$$\begin{aligned} m(t_f) &= k' e^{(b_2 - d_2)t_f} \approx 6.0 \times 10^9 \\ \sigma(t_f) &= \sqrt{k' \frac{b_2 + d_2}{b_2 - d_2} e^{(b_2 - d_2)t_f} \left(e^{(b_2 - d_2)t_f} - 1\right)} \approx 9.1 \times 10^5 \end{aligned} \tag{G11}$$

With analogous argumentation to the decreasing population, we conclude that the growth is approximately deterministic and the SFS of preexisting mutations retains its k^{-2} tail.

Newly emerging mutations are restricted to small frequencies. In contrast to decreasing and the constant populations, newly emerging mutations grow in their abundance not only to due to genetic drift. However, since the cancer is usually detected at very large sizes, and preexisting site frequencies grow at the same rate, it is highly unlikely that newly emerging mutations grow to 1% of the population. With the estimated birth and death rate, we have mean and standard deviation for the abundance of a newly emerging mutation growing for time $t_f = 180$ days,

$$\begin{aligned} m(t_f) &= e^{(b_2 - d_2)t_f} \approx 6.0 \\ \sigma(t_f) &= \sqrt{\frac{b_2 + d_2}{b_2 - d_2} e^{(b_2 - d_2)t_f} \left(e^{(b_2 - d_2)t_f} - 1\right)} \approx 28.7. \end{aligned} \tag{G12}$$

Although the standard deviation is very large compared to the mean, the likelihood to reach observable size $k_{\min} \approx 6.0 \times 10^9$ is again virtually zero.

H Proof of Theorem 2

Part 1: We prove eqn. (17) building up on the proof of Theorem 1. Restricted (but not conditioned) on survival of the entire population $\{Z_0(t) > 0\}$, the expected number of mutations that emerged in the

time interval $[t', t'+dt']$ and survive in the remaining time $t-t'$ is

$$\begin{aligned} E\left[\left(B^{(new)}(t'+dt') - B^{(new)}(t)\right) 1_{\{Z_0(t)>0\}}\right] &= \sum_{k\geq 1} E\left[\left(S_k^{(new)}(t'+dt') - S_k^{(new)}(t)\right) 1_{\{Z_0(t)>0\}}\right] \\ &= bE[\Omega]E[Z_0(t')] \sum_{k\geq 1} p(1\to k, t-t') \\ &= bE[\Omega]E[Z_0(t')]\left(1-p(1\to 0, t-t')\right). \end{aligned} \tag{H1}$$

In the first equality, we used that $B^{(new)} = \sum_{k\geq 1} S_k^{(new)}$ together with linearity of the expectation. The second equality is equivalent to the first equation in the proof of Theorem 1 taken from Gunnarsson *et al.* (2021). Again, Ω is the number of mutations generated in a division such that $E[\Omega] = 2m$ and the expected population size is $E[Z_0(t')] = N(t') = N_0 e^{(b-d)t'}$. After conditioning the total population to survive time t, we have

$$E\left[\left(S^{(new)}(t'+dt') - S^{(new)}(t)\right) | Z_0(t)>0\right] = \frac{1}{1-p(N_0\to 0,t)} 2mbN(t')(1-p(1\to 0, t-t')). \tag{H2}$$

We integrate over the time interval of interest $t'\in[0,t]$ giving us

$$E[B^{(new)}] = \frac{1}{1-p(N_0\to 0,t)} \int_0^t 2mbN(t')(1-p(1\to 0,t-t'))dt', \tag{H3}$$

which makes the proof complete.

The expected number of divisions is

$$E[R^{(new)}] = E[B^{(new)}]/m = \frac{1}{1-p(N_0\to 0,t)} \int_0^t 2bN(t')(1-p(1\to 0,t-t'))dt'. \tag{H4}$$

Part 2: Next, we prove eqn. (18). Analogously to the proof of the second part of Theorem 1, we start by writing the initial BWD as $W_k^{(init)}$, label its L branches with index $i = 1, 2, ...L$ and their branch widths with k_i. During time t, branches may go extinct and we count branches leading to at least one living cell that is

$$R^{(pre)} = \sum_{i=1}^{L} 1_{\{k_i\to k>0\}} = \sum_{k'=1}^{N_0} \sum_{j=1}^{W_{k'}^{(init)}} 1_{\{k_j\to k>0\}}. \tag{H5}$$

Here, $1_{\{k_i\to k>0\}} = 1$ if branch i of initial width k_i grows to size $k>0$ and $1_{\{k_i\to k\}} = 0$ otherwise. Again, we used that the L branches are partitioned into N_0 classes $\{W_{k'}^{(init)}\}_{k'=1}^{N_0}$ according to their initial width k'.

Taking the expectation and using the same steps as in the proof of Theorem 1, we have

$$\begin{aligned} E[B^{(pre)}] &= E\left[\sum_{k'=1}^{N_0} \sum_{j=1}^{W_{k'}^{(init)}} 1_{\{k_j\to k>0\}}\right] \\ &= \sum_{k'=1}^{N_0} E\left[\sum_{j=1}^{W_{k'}^{(init)}} 1_{\{k_j\to k>0\}}\right] \\ &= \sum_{k'=1}^{N_0} E[W_{k'}^{(init)}]\,(1-p(k'\to 0,t)). \end{aligned} \tag{H6}$$

We substitute $E[R^{(pre)}] = E[B^{(pre)}]/m$ and $E[W_k^{(init)}] = E[S_k^{(init)}]/m$ and condition the entire population on survival such that

$$E[B^{(pre)}] = \frac{1}{1 - p(N_0 \to 0, t)} \sum_{K=1}^{N_0} E[S_{k'}^{(init)}]\,(1 - p(k' \to 0, t)), \tag{H7}$$

which is the desired result.

I The total mutational burden at detection

Pure-birth process If we consider the fixed-size limit, we know that there are exactly $N_d - 1$ divisions that a population must undergo to grow form 1 cell to N_d cells. Thus, the expected tMB is

$$E[B^{(det)}] = (N_d - 1)2m \approx 2N_d m \tag{I1}$$

Furthermore, since each division comes with $\mu_i \sim \text{Poiss}(m)$ mutations, we know that the tMB is Poisson distributed

$$B^{(det)} \sim \text{Poiss}(2N_d m). \tag{I2}$$

If there were u_0 mutations in the ancestor cell, one simply needs to add them as constant outside the Poisson distribution, $B^{(det)} \sim \text{Poiss}(2N_d m) + u_0$.

In the fixed-time condition, we consider a population growing time t_d at which we expect population size $N_d = E[Z_0(t_d)]$. This translates into an expected number of $N_d(t_d) - 1$ divisions similar to the fixed-size expectation. However, the distribution of $B^{(det)}$ is not Poisson distributed anymore. The number of divisions differs between realizations thus leading to additional stochastic effect.

Birth-death process Following Theorem 2, starting from a single cell with no mutations that grows for time t_d and is conditioned to survive, we have

$$\begin{aligned} E[B^{(new)}] &= \frac{1}{1 - p(k \to 0, t_d)} \int_0^{t_d} b_1 2mN(t) \times (1 - p(1 \to 0, t_d - t)))dt \\ &= -2m \frac{e^{(b_1 - d_1)t_d}}{1 - p(k \to 0, t_d)} \frac{b_1}{d_1} \log\left(\frac{b_1 - d_1}{b_1 - d_1 e^{-(b_1 - d_1)t_d}}\right) \\ &= 2mN_d \frac{b_1}{d_1} \log\left(\frac{b_1}{b_1 - d_1} \frac{1}{1 - p(1 \to 0, t_d - t)}\right). \end{aligned} \tag{I3}$$

We can further manipulate the expression to show agreement with ref. Gunnarsson *et al.* (2021). We have

$$\begin{aligned} E[B^{(new)}] &= -2mN_d \frac{b_1}{d_1} \log\left(\frac{b_1 - d_1}{b_1 - d_1(N_d\sigma + \rho)^{-1}}\right) \\ &= -2mN_d \frac{b_1}{d_1} \log\left(\sigma + \frac{\rho}{N_d}\right) \\ &\sim -2mN_d \frac{\log(\sigma)}{\rho} \qquad \text{for } N_d \to \infty, \end{aligned} \tag{I4}$$

where we used that $N_d = \frac{1}{1-p(1 \to 0, t_d)} e^{(b_1 - d_1)t_d} = \frac{e^{(b_1-d_1)t} + \rho}{\sigma}$.

If there be u_0 mutations preexisting in the first cell, they simply need to be added as a constant such that total tMB is $E[B^{(tot)}] = E[B^{(new)}] + u_0$.

J The total mutational burden after treatment with homogeneous response

We compute the expected tMB after treatment with homogeneous treatment response. As initial condition, we consider fixed size N'_d and the expected SFS at detection. For the initial SFS, we approximate the fixed-size expectation of the SFS with the fixed-time expectation of the SFS by replacing the expected size $N_d = \tilde{N}(t_d)$ with the fixed-size N'_d in eqn. (9). We denote the treatment time by t_f after which the population conditioned on survival has expected size $N_f = \frac{1}{1-p(N'_d \to 0)} N'_d e^{(b_2-d_2)t_f}$. Since the extinction probability appears in the final expressions, it is convenient to write $\alpha(t)$ for $p(1 \to 0, t)$, and $\alpha^a(t)$ for $p(a \to 0, t)$.

J.1 Decreasing cell populations

We consider decreasing cell populations characterized by $b_2 < d_2$ and $N_f < N'_d$.

Newly emerging mutations: Following Theorem 2, we have

$$\begin{aligned}
E[B^{(new)}] &= \frac{1}{1-\alpha^{N'_d}(t_f)} \int_0^{t_f} 2mb_2 N'_d e^{(b_2-d_2)t} \left(1-\alpha(t_f - t)\right) dt \\
&= -2mN_f \frac{b_2}{d_2} \log\left(\frac{b_2-d_2}{b_2 - d_2 e^{-(b_2-d_2)t_f}}\right) \\
&= 2mN_f \frac{b_2}{d_2} \log\left(\frac{b_2}{b_2-d_2} - e^{-(b_2-d_2)t_f}\frac{d_2}{b_2-d_2}\right) \\
&= 2mN_f \frac{b_2}{d_2} \log\left(\frac{b_2}{b_2-d_2} - \frac{1}{1-\alpha(t_f)}\frac{N'_d}{N_f}\frac{d_2}{b_2-d_2}\right),
\end{aligned} \tag{J1}$$

For $t_f \to \infty$, the expression coincides with the the tMB at detection. In particular, it is independent of the initial population size N'_d. This makes sense. Newly emerging mutations are only short-lived and the major contributions to $B^{(new)}$ are very recent mutations.

Preexisting mutations: Following Theorem 2, we have

$$E[B^{(pre)}] = \frac{1}{1-\alpha^{N'_d}(t_f)} \sum_{k=1}^{N'_d} E[S_k^{(det)}] \left(1-\alpha^k(t_f)\right). \tag{J2}$$

Here, $\alpha(t)$ is given by eqn. (1) parameterized with b_2 and d_2. By construction, the initial SFS is the SFS at detection whose expectation is given by eqn. (9).

J.2 Constant cell populations

Next, we consider cell populations that remain approximately constant characterized by $b_2 = d_2$ and $N'_d \approx N_f$.

Newly emerging mutations: Following Theorem 2, we have

$$\begin{aligned} E[B^{(new)}] &= \frac{1}{1-\alpha^{N'_d}(t_f)} \int_0^{t_f} (2mb_2N'_d)\ (1-\alpha(t_f-t))\ dt \\ &= 2mN_f \log(1+b_2t_f)\,. \end{aligned} \tag{J3}$$

Preexisting mutations: Following Theorem 2, we have

$$E[B^{(pre)}] = \frac{1}{1-\alpha^{N'_d}(t_f)} \sum_{k=1}^{N'_d} E[S_k^{(det)}]\left(1-\alpha^k(t_f)\right), \tag{J4}$$

Again, $\alpha(t)$ is given by eqn. (1) parameterized with b_2 and d_2. The initial SFS is given by eqn.(9).

J.3 Increasing cell populations

Eventually, we consider increasing cell populations characterized by $b_2 > d_2$ and $N_f > N'_d$.

Newly emerging mutations: The expressions for increasing populations coincide with the ones for decreasing populations such that

$$\begin{aligned} E[B^{(new)}] &= \frac{1}{1-\alpha^{N'_d}(t_f)} \int_0^{t_f} 2mb_2N'_d e^{(b_2-d_2)t}\ (1-\alpha(t_f-t))\ dt \\ &= 2mN_f\frac{b_2}{d_2}\log\left(\frac{b_2}{b_2-d_2} - \frac{1}{1-\alpha(t_f)}\frac{N'_d}{N_f}\frac{d_2}{b_2-d_2}\right). \end{aligned} \tag{J5}$$

In the limit $\frac{N'_d}{N_f} \to 0$, the expression is independent

Preexisting mutations: Following Theorem 2, we have

$$E[B^{(pre)}] = \frac{1}{1-\alpha^{N'_d}(t_f)} \sum_{k=1}^{N'_d} E[S_k^{(det)}]\left(1-\alpha^k(t_f)\right). \tag{J6}$$

Once more, $\alpha(t)$ is given by eqn. (1) parameterized with b_2 and d_2. The initial SFS is given by eqn. (9).

J.4 Approximation for preexisting mutations

We resolve the sum for $E[B^{(pre)}]$ using diverse approximation. In this section, the extinction probability will always be the extinction probability after time t_f such that we write $\alpha = \alpha(t_f)$ that are parameterized with b_2 and d_2.

Consider $E[S_k^{(det)}] \sim \frac{1}{k^2}$ as expected SFS at detection. For large site frequencies k, the SFS at detection is approximately $E[S_k^{(det)}] \approx \frac{b_1}{b_1-d_1} 2mN'_d \frac{1}{k^2}$. Using this expression for $E[B^{pre}]$ and with help of *Wolfram Mathematica*, we obtain

$$\begin{aligned} E[B^{(pre)}] &= \frac{1}{1-\alpha^{N'_d}(t_f)} \sum_{k=1}^{N'_d} \frac{b_1}{b_1-d_1} 2mN'_d \frac{1}{k^2}\left(1-\alpha^k\right) \\ &= \frac{1}{1-\alpha^{N'_d}(t_f)} \frac{b_1}{b_1-d_1} 2mN'_d \left(\alpha^{N'_d+1}\Phi(\alpha,2,N'_d+1) + H^{(2)}_{N'_d} - \mathrm{Li}_2(\alpha)\right), \end{aligned} \tag{J7}$$

where $\Phi(z,s,a) = \sum_{j=0}^{\infty} \frac{z^j}{(j+a)^s}$ is the Lerch phi, $\mathrm{Li}_n(z) = \sum_{j=0}^{\infty} \frac{z^j}{j^n}$ is the polylogarithm and $H_n^{(2)} = \sum_{j=1}^{n} \frac{1}{j^n}$ are generalized harmonic numbers. For sufficiently large N'_d, the first term in the parentheses becomes very small and we are left with

$$E[B^{(pre)}] \approx \frac{b_1}{b_1-d_1} 2mN'_d \left(H^{(2)}_{N'_d} - \mathrm{Li}_2(\alpha)\right). \tag{J8}$$

Notably, the $H^{(2)}_{N'_d}$ is time-independent and positive whereas $-\mathrm{Li}_2(\alpha)$ is time-dependent and negative. We can interpret the first term as the number of mutations at detection which we can also obtain directly by summing over $\frac{1}{k^2}$. Then, the second term describes the number of mutations that are lost over time.

Consider $E[S_k^{(det)}] \sim \frac{1}{k^2+k}$ as expected SFS at detection. Most mutations occur at low abundance where $E[S_k^{(det)}] \sim \frac{1}{k}$ is not valid. To get one step closer to the exact expected SFS at detection, we next consider $E[S_k^{(det)}] \approx 2mN'_d \frac{b_1}{b_1-d_1} \frac{1}{k^2+k}$. Using *Wolfram Mathematica*, we obtain

$$\sum_{k}^{N'_d} \frac{1}{k^2+k}(1-\alpha)^k = \alpha^{N'_d+1}\Phi(\alpha,1,N'_d+1) - \alpha^{N+1}\Phi(\alpha,1,N'_d+2) + \frac{(\alpha-1)\log(1-\alpha)}{\alpha} - \frac{1}{N'_d+1} \tag{J9}$$

For large N'_d, the first two terms and the last term become very small leaving us with

$$\sum_{k}^{N'_d} \frac{1}{k^2+k}(1-\alpha)^k \approx \frac{(\alpha-1)\log(1-\alpha)}{\alpha}. \tag{J10}$$

Using this expression to compute the tMB of preexisting mutations, we obtain

$$E[B^{(pre)}] \approx \frac{b_1}{b_1-d_1} 2mN'_d \frac{(\alpha-1)\log(1-\alpha)}{\alpha}. \tag{J11}$$

Heuristic adjustment at $t_f = 0$. At $t_f = 0$, the extinction probability is $\alpha = 0$, and

$$\lim_{\alpha\to 0} \frac{(\alpha-1)\log(1-\alpha)}{\alpha} = 1 \tag{J12}$$

such that $E[B^{(pre)}] \approx \frac{b_1}{b_1-d_1} 2mN'_d$ at time $t_f = 0$. This is not accurate for the general birth-death process as we know from the section on the tMB at detection. Requiring a correct result at time $t_f = 0$, we make a heuristic correction and write

$$E[B^{(pre)}] \approx E[B^{(det)}] \frac{(\alpha-1)\log(1-\alpha)}{\alpha}, \tag{J13}$$

where $E[B^{(det)}]$ is given by eqn. (19).

K Proof of Theorem 3

Part 1: We prove eqn. (24). Taking the expectation of the scMB described by eqn. (23), we have

$$\begin{aligned} E[M_j] &= E\left[\sum_{l=1}^{\infty}\sum_{m=1}^{D_l} 1_{\{\sum_{n=1}^{l}\mu_n=j\}}\right] \\ &= \sum_{l=1}^{\infty} E\left[\sum_{m=1}^{D_l} 1_{\{\sum_{n=1}^{l}\mu_n=j\}}\right] \\ &= \sum_{l=1}^{\infty} E[D_l]E\left[1_{\{\sum_{n=1}^{l}\mu_n=j\}}\right] \\ &= \sum_{l=1}^{\infty} E[D_l]\,\mathrm{Pr}\left(\sum_{n=1}^{l}\mu_n=j\right) \\ &= \sum_{l=1}^{\infty} E[D_l]\frac{(lm)^k e^{-lm}}{k!}. \end{aligned} \tag{K1}$$

Part 2: Next, we prove eqn. (25). We denote the number of cells with l divisions at time t by $D_l(t)$. We follow the derivation of Williams *et al.* (2018). We start from a population with N_0 cells at time $t = 0$ such that $D_0(t = 0) = N_0$. Considering only the mean behaviour, we write the following differential equations for D_l

$$\begin{aligned} \frac{D_0}{dt} &= -(b+d)D_0 \\ \frac{D_l}{dt} &= -(b+d)D_l + 2bD_{l-1}, \end{aligned} \tag{K2}$$

which has solutions

$$\begin{aligned} D_0(t) &= N_0 e^{-(b+d)t} \\ D_l(t) &= \frac{(2bt)^l}{l!}D_0(t) \end{aligned} \tag{K3}$$

The expected population size after time t is $N = N_0 e^{(b-d)t}$, which leads to the desired result.

L Average number of divisions in the population

L.1 Increase of the average generation per division

The following derivation was motivated by Moeller *et al.* (2024). Consider a population of N cells, where the number of divisions that each cell has undergone are saved in a vector $\vec{x} = [x_1, ..., x_N]$ with mean $\bar{x} = \frac{1}{N}\sum_{i=1}^{N} x_i$. After one division, the population has increased to size $N + 1$ and we have division vector $\vec{y} = [y_1, ..., y_{N+1}]$ with mean $\bar{y} = \frac{1}{N+1}\sum_{i=1}^{N+1} y_i$. Let us denote the index of the dividing cell by j

such that $x_i = y_i$ for $i \neq j$ and $y_j = y_{N+1} = x_j + 1$. Then, we have

$$\begin{aligned}\bar{y} &= \frac{1}{N+1}\sum_{i=1}^{N+1} y_i \\ &= \frac{1}{N+1}\left(N\bar{x} + x_j + 2\right)\end{aligned} \tag{L1}$$

If we now average over many realizations, then

$$\begin{aligned}E[\bar{y}] &= E\left[\frac{N\bar{x} + x_j + 2}{N+1}\right] \\ &= \frac{NE[\bar{x}] + E[x_j] + 2}{N+1}\end{aligned} \tag{L2}$$

Now, note that

$$E[\bar{x}] = E\left[\frac{1}{N}\sum_{i=1}^{N} x_i\right] = \frac{1}{N}NE[x_i] = E[x_i], \tag{L3}$$

such that we have

$$E[\bar{y}] = E[\bar{x}] + \frac{2}{N+1}. \tag{L4}$$

Thus, the average increase for going from size N to size $N+1$ is given by $\frac{2}{N+1}$.

L.2 Fixed-size solution in the pure-birth process

For a pure-birth process starting from 1 cell with 0 divisions, we have $N-1$ divisions to grow to size N where the i-th division increases the generation average by $\frac{1}{1+i}$ such that the expected average number of division reads

$$E[\bar{l}] = \frac{2}{1+1} + \frac{2}{2+1} + \frac{2}{3+1}... = \sum_{i=1}^{N-1}\frac{2}{i+1} = 2(H_N - 1). \tag{L5}$$

More generally starting from N_0 cells with 0 divisions growing to size N, we have

$$E[\bar{l}] = \frac{2}{N_0+1} + \frac{2}{N_0+2} + ... = \sum_{i=1}^{N-1}\frac{2}{N_0+i} = 2(H_N - H_{N_0}). \tag{L6}$$

L.3 Fixed-time solution in the pure-birth process

In the spirit of the proof of Theorem 1 and 2 but without strict proof, we write the increase of the average number of divisions in time interval $[t', t+, dt']$ as

$$E[\bar{l}(t'+dt') - \bar{l}(t')] = bN(t')\frac{2}{N(t')+1}. \tag{L7}$$

We restrict ourselves to the pure-birth process as conditioning on survival complicates the derivation. After summing over the entire time interval $[0, t]$, we have

$$E[\bar{l}] = 2b\int_0^t \frac{N(t')}{N(t')+1}\,dt'. \tag{L8}$$

Taking $N(t) = N_0 e^{bt}$, this reads

$$E[\bar{l}] = 2bt_{\text{corr}} \quad \text{with} \quad t_{\text{corr}} = \frac{\log\left(bN_0e^{bt} + b\right) - \log(b(N_0+1))}{b}. \tag{L9}$$

L.4 Comparison between mean-field solution and exact solution in the pure-birth process

Fixed-time solutions: Interestingly, even as time goes to infinity, there is a constant off-set between eqn. L9 and the mean field solution $E[\bar{l}^{(mf)}] = 2bt$ from Theorem 3. We have

$$\lim_{t\to\infty} E[\bar{l}^{(mf)}] - E[\bar{l}^{(exact)}] = 2\ln\left(\frac{N_0+1}{N_0}\right) \tag{L10}$$

For $N_0 = 1$, this yields a constant off-set of $\ln(4) \approx 1.386$, and for $N_0 \to \infty$, the difference is vanishing and we have $E[\bar{l}^{(mf)}] = E[\bar{l}^{(exact)}]$.

Fixed-size solutions: We obtain a similar result in the fixed size condition. We transform the mean-field fixed-time solution into a fixed-size solution using according to Appenxid C such that

$$E[\bar{l}^{(mf)}|N] = \int_0^\infty (2bt)\, f_T(t)\, dt = 2(\gamma + \ln(N)). \tag{L11}$$

We note that $f_T(t)$ assumes that $N_0 = 1$. To compare this to the exact fixed-size solution, we approximate the harmonic number with its asymptotic limit $H_N = \gamma + \ln(N) + O\left(\frac{1}{N}\right)$ giving us

$$E[\bar{l}^{(exact)}|N] = 2(\gamma + \ln(N) - 1) + O\left(\frac{1}{N}\right) \tag{L12}$$

such that

$$E[\bar{l}^{(mf)}|N] - E[\bar{l}^{(exact)}|N] = 2 + O\left(\frac{1}{N}\right). \tag{L13}$$

The asymptotic solution differs from the exact solution by 2, which agrees with the correction term computed for the critical branching process from Cheek and Johnston (2023). Further, computer simulations show that the correction term 2 is in good agreement with the birth-death process starting from a single cell.

M Size distribution of resistant clones

We consider sensitive cells growing with rates $b_{s,1}$ and $d_{s,1}$ in the absence of treatment and $b_{s,2}$ and $d_{s,2}$ during treatment. During division of sensitive cells, each daughter cells acquires resistance mutations at rate $\nu \ll 1$. Resistant cells grow with rates $b_{r,1}$ and $d_{r,1}$ in the absence of treatment and $b_{r,2}$ and $d_{r,2}$ during treatment. Since the generation of resistance mutations is really small and our model does not include any other interactions between sensitive and resistant cells, we can approximate the growth of sensitive cells using growth rates $b_{s,1} - d_{s,1}$ and $b_{s,2} - d_{s,2}$ rather than $b_{s,1} - d_{s,1} - \nu$ and $b_{s,2} - d_{s,2} - \nu$.

We further neglect the chance that more than one resistance mutation emerge in one daughter during one division. Such events come with probability of the order ν^2. Using these approximations, the CSD can be computed in the same way as the SFS.

Clone size distribution at detection. Before treatment, resistant clones are generated at rate $2\nu e^{(b_{s,1}-d_{s_1})t}$ and grow to size κ with probability $p(1 \to \kappa, t_d - t)$. Following Theorem 1, we have

$$E[\Theta_\kappa^{(det)}] = \frac{1}{1-p(1\to 0, t_d)} \int_0^{t_d} 2\nu e^{(b_{s,1}-d_{s,1})t} p(1 \to \kappa, t_d - t)\, dt. \tag{M1}$$

Whereas the term $p(1 \to \kappa, t_d - t)$ described the growth of resistant clones and thus must be parameterized with $b_{r,1}$ and $d_{r,1}$, the term $\frac{1}{1-p(1\to 0,t_d)}$ describes the conditioning on survival of the entire population that is appropriately parameterized with $b_{s,1}$ and $d_{s,1}$.

If sensitive and resistant cells share the same growth parameters in the absence of treatment, i.e. $b_{s,1} = b_{r,1} := b_1$ and $d_{s,1} = d_{r,1} := d_1$, then the CSD takes the same form as the SFS from eqn. (9), namely

$$\begin{aligned} E[\Theta_\kappa^{(det)}] &= 2\nu N_d \int_0^{1-1/N_d} \left(1 - \frac{d_1}{b_1} y\right)^{-1} (1-y) y^{\kappa-1}\, dy \\ &\to 2\nu N_d \sum_{l=0}^{\infty} \frac{\left(\frac{d_1}{b_1}\right)^l}{(\kappa+l)(\kappa+l+1)} \quad \text{for } N_d \to \infty \\ &\to 2\nu N_d \frac{b_1}{b_1 - d_1} \frac{1}{\kappa(\kappa+1)} \quad \text{for } \kappa \to \infty. \end{aligned} \tag{M2}$$

Clone size distribution after treatment. The CSD after treatment for time t_f is also obtained by adapting Theorem 1. For newly emerging mutations, the relevant mutation generation comes from sensitive cells. Starting from N'_d cells, the mutation generation is described by $2\nu b_{s,2} N'_d e^{(b_{s,2}-d_{s,2})t}$. We drop the term $\frac{1}{1-p(N'_d \to 0, t_f)}$ for conditioning on survival such that

$$E[\Theta_\kappa^{(new)}] = \int_0^{t_f} b_{s,2} 2\nu N'_d e^{(b_{s,2}-d_{s,2})t} p(1 \to \kappa, t_f - t)\, dt. \tag{M3}$$

The term $p(1 \to \kappa, t_f - t)$ described the growth of resistant clones and thus must be parameterized with $b_{r,2}$ and $d_{r,2}$. For the CSD of clones preexisting to treatment, we again drop the conditioning on survival and obtain

$$E[\Theta_\kappa^{(pre)}] = \sum_{i=1}^{N'_d} E[\Theta_{\kappa'}^{(det)}] p(\kappa' \to \kappa, t_f), \tag{M4}$$

with $p(\kappa' \to \kappa, t_f)$ parameterized with $b_{r,2}$ and $d_{r,2}$.

To justify dropping the conditioning on survival, we note that we start from an initially large number of sensitive and resistant cells. First, it is highly unlikely that resistant cells go extinct. Second, the relevant mutational input for the CSD comes from the sensitive cell population. For small times t, the average growth of sensitive cells with conditioning on survival is $\tilde{N}_s(t) = \frac{1}{1-\alpha(t)} N'_d e^{(b_{s,2}-d_{s,2})t}$ that is well approximated by the average growth of sensitive cells without conditioning $N_s(t) = N'_d e^{(b_{s,2}-d_{s,2})t}$. For larger t, when the sensitive cell population approaches the extinction threshold, there will be so few sensitive cells that the generation of new clones will be negligible, $b_{s,2} 2\nu N'_d e^{(b_{s,2}-d_{s,2})t} \to 0$. Together, this allows us to drop the term $\frac{1}{1-p(N'_d \to 0, t_f)}$ in Theorem 1 while using $N_s(t)$ rather than $\tilde{N}_s(t)$ for the generation of new mutations.

N Resistant subpopulation versus largest resistant clone

In growing populations, it is apparent that the random timing of mutations plays an important role in determining the mutant population size. Here, we ask whether the largest resistant clone dominates the total resistant subpopulation. First, we compute the arrival time of the first and second mutant and note that the second arriving mutant may outgrow the first due to genetic drift. Secondly, we derive the probability distributions for the size of the largest clone and compare this to the probability distribution for the total number of mutant cells.

We focus on clone sizes at detection since a resistant clone dominating at detection will also dominate after treatment. Therefore, we consider a birth-death process with birth rate b_1 an death rate d_1 growing from a single cell up to size N_d, while each daughter cell acquires resistance mutation at rate $\nu \ll 1$ during a division. The mutation rate per division is then $\tilde{\nu} = 2\nu$.

N.1 Arrival time of resistant clones

In order to condition on survival of genetic drift, we can adapt the mutation rate $\tilde{\nu} \to \tilde{\nu}\sigma$, where $\sigma = \frac{b_1 - d_1}{b_1}$ is the probability to survive drift in the linear birth-death process (Durrett 2015).

Considering deterministic growth of the entire population with random mutations, the number of acquired resistance mutations follows an in-homogeneous Poisson process such that the resistance mutations acquired in the time interval $[\tau_1, \tau_2]$ is given by

$$P_k = \frac{e^{-\lambda}\lambda^k}{k!} \qquad \text{with } \lambda = \int_{\tau_1}^{\tau_2} b_1 \tilde{\nu} e^{(b_1-d_1)t}\, dt. \tag{N1}$$

To compute the arrival time of the first mutant, we set $\tau_1 = 0$ and $\tau_2 = t_1$. The probability that no mutations were acquired up to time t_1 is P_0. We identify $1 - P_0$ as the cumulative density function for the arrival time of the first surviving mutant. Taking the derivative of the cumulative density function, we obtain the probability density function for T_1 that reads

$$f_{T_1}(t_1) = \frac{d(1-P_0)}{dt_1} = b_1 \tilde{\nu} e^{(b_1-d_1)t_1 - \frac{b_1\tilde{\nu}}{b_1-d_1} e^{(b_1-d_1)t_1}}. \tag{N2}$$

Similar results were previously reported by making approximations on the fully stochastic model (e.g. chapter 5 in (Durrett 2015)).

Given that the first resistant clone arose at $T_1 = t_1$, the probability that no second mutations occurs until time t_2 is P_0 but with $\tau_1 = t_1$ and $\tau_2 = t_2$. Analogously to T_1, we compute the probability density function for T_2,

$$f_{T_2}(t_2 | T_1 = t_1) = \frac{d(1-P_0)}{dt_2} = b_1 \tilde{\nu} e^{(b_1-d_1)t_2 - \frac{b_1\tilde{\nu}}{b_1-d_1}\left(e^{(b_1-d_1)t_2} - e^{(b_1-d_1)t_1}\right)} 1_{[t_1,\infty]}(t_2). \tag{N3}$$

The unconditional density of T_2 is obtained by marginalizing out T_1,

$$\begin{aligned} f_{T_2}(t_2) &= \int_0^\infty f_{T_2}(t_2|T_1 = t_1) f_{T_1}(t_1)\, dt_1 \\ &= \frac{b_1^2 \tilde{\nu}^2}{b_1 - d_1} \left(e^{(b_1-d_1)t_2} - 1 \right) e^{(b_1-d_1)t_2 - \frac{b_1 \tilde{\nu}}{b_1 - d_1} e^{(b_1-d_1)t_2}} \\ &\approx \frac{b_1^2 \tilde{\nu}^2}{b_1 - d_1} e^{2(b_1-d_1)t_2 - \frac{b_1 \tilde{\nu}}{b_1-d_1} e^{(b_1-d_1)t_2}}. \end{aligned} \tag{N4}$$

Inverse cumulative density function: For Fig. 5a, we generate random numbers for T_1 and T_2. Therefore, we use inverse transformation sampling, which requires the inverse distribution function. For T_1, we have

$$t_1 = F_{T_1}^{-1}(u) = \frac{1}{b_1 - d_1} \log \left(\frac{(b_1 - d_1)) \log \left(\frac{1}{1-u} \right)}{b_1 \tilde{\nu}} + 1 \right). \tag{N5}$$

For T_2 conditioned on $T_1 = t_1$, we have

$$t_2 = F_{T_2|T_1}^{-1}(u) = \frac{1}{b_1 - d_1} \log \left(e^{(b_1-d_1)t1} - \frac{(b_1 - d_1) \log(1-u)}{b_1 \tilde{\nu}} \right). \tag{N6}$$

N.2 Size of the resistant subpopulation

In the limit $\tilde{\nu}N \gg 1$, the probability to have R resistant cells is described by a Landau distribution (Kessler and Levine 2013, 2015) of the form

$$P(R) = \frac{b-d}{b} \frac{1}{\tilde{\nu}N} P_{\text{Landau}} \left(\frac{b-d}{b} \frac{R}{\tilde{\nu}N} - \ln(\tilde{\nu}N) \right). \tag{N7}$$

For $\tilde{\nu}N \sim 1$, the solution is slightly more complicated but also given by Kessler and Levine (2015). The fixed-time solution was computed by Antal and Krapivsky (2011) and some asymptotic solutions including selection are derived in by Nicholson *et al.* (2023). However, we focus on the case $\tilde{\nu}N \gg 1$.

N.3 Size of the largest clone

We denote the number of clones with size κ by Θ_κ such that $\{\Theta_\kappa\}_{\kappa=1}^N$ is the clone size distribution (CSD) similar to the definition of the SFS in the main text. As explained in the main text, the CSD is described by the same expression as the SFS. Since we are interested in the largest clone only that is unlikely to go extinct (should the population continue growing indefinitely), it is sufficient to consider skeleton spectrum (i.e. clones that will never go extinct) in which case the CSD takes the form described in Proposition 1 from Gunnarsson *et al.* (2021), namely

$$\begin{aligned} E[\Theta_\kappa^{(skel)}] &\approx \frac{b_1}{b_1 - d_1} \tilde{\nu} N_d \left(1 - \frac{1}{N_d}\right)^\kappa \left(\frac{1}{\kappa(\kappa+1)} + \frac{1}{N} \frac{1}{\kappa} \right) \\ &\sim \frac{b_1}{b_1 - d_1} \tilde{\nu} N_d \frac{1}{\kappa(\kappa+1)} \qquad \text{for} \quad N_d \to \infty \end{aligned} \tag{N8}$$

For simplicity, we take $\kappa \in [1, \infty)$ on a continuous scale and approximate $\frac{1}{\kappa^2+\kappa} \approx \frac{1}{\kappa^2}$. Then, the skeleton CSD can written as product of an amplitude A and probability density $f(\kappa)$,

$$E[\Theta_\kappa^{(skel)}] = Af(\kappa) \quad \text{with } A = \frac{b_1}{b_1 - d_1}\tilde{\nu}N_d \text{ and } f(\kappa) = \frac{1}{\kappa^2}. \tag{N9}$$

The amplitude is interpreted as the number of mutations with infinite lineage (Gunnarsson *et al.* 2021). If we take this number as fixed, then the largest clone size can be understood as order statistic. In other words, the largest clone size $\tilde{\kappa}$ is the largest number of A clone sizes that are sampled from $f(\kappa)$.

Assuming independence between clone sizes, the cumulative density function for $\tilde{\kappa}$ is simply the A-th power of the cumulative density function for κ (David and Nagaraja 2004). The cumulative density function for κ reads $F(\kappa) = 1 - \frac{1}{\kappa}$ such that

$$F(\tilde{\kappa}) = \left(1 - \frac{1}{\tilde{\kappa}}\right)^A \tag{N10}$$

and the probability density is

$$f(\tilde{\kappa}) = \frac{dF}{d\tilde{\kappa}} = \frac{A}{\tilde{\kappa}^2}\left(1 - \frac{1}{\tilde{\kappa}}\right)^{A-1}. \tag{N11}$$

N.3.1 Comparison with Cheek and Antal (2018)

Besides our study, Cheek & Antal also investigated the size distribution of the largest clone (Proposition 4.6 in ref. (Cheek and Antal 2018)). A closed-form expression is given for the case $d_{r,1} = 0$. Keeping their notation, it is stated that

$$\Pr(M^* \le k) = e^{-\frac{\theta}{\lambda_A(k+1)}}. \tag{N12}$$

We transform parameters into our notation through $\theta \to b_s\tilde{\nu}N_d$, $\lambda_A \to b_{s,1} - d_{s,1}$ and $k \to \kappa$ after which we have cumulative distribution

$$F_{CA}(\tilde{\kappa}) = \Pr(M^* \le \tilde{\kappa}) = e^{-\frac{b_{s,1}\tilde{\nu}N_d}{(b_{s,1}-d_{s,1})(k+1)}}. \tag{N13}$$

The probability density is then

$$f_{CA}(\tilde{\kappa}) = \tag{N14}$$

To note the similarity to our prediction, we write $\sigma_s = \frac{b_{s,1}-d_{s,1}}{b_{s,1}}$ and note that

$$\begin{aligned} F_{CA}(\tilde{\kappa}) &= \lim_{\sigma_s^{-1}\tilde{\nu}N_d \to \infty}\left(1 - \frac{\sigma_s^{-1}\tilde{\nu}N_d \times \frac{1}{\tilde{\kappa}+1}}{\sigma_s^{-1}\tilde{\nu}N_d}\right)^{\sigma_s^{-1}\tilde{\nu}N_d} \\ &\approx \left(1 - \frac{1}{\tilde{\kappa}+1}\right)^{\sigma_s^{-1}\tilde{\nu}N_d}, \end{aligned} \tag{N15}$$

where we assume that the resistant subpopulation consists of many clones in the approximation. After identifying $A = \sigma_s^{-1}\tilde{\nu}N_d$, this expression coincides with our expression in eqn. (N10) up to a difference in $\tilde{\kappa} \leftrightarrow \tilde{\kappa} + 1$.

O Site frequency spectrum upon emergence of resistance

Following the main text, we separate between mutations that occur in sensitive cells and mutations that occur in resistant cells. The sum of both contributions will make the total SFS,

$$S_k = S_k^S + S_k^R \quad \text{with} \quad S_k^R = \sum_{i=1}^{K} S_k^{R,i}. \tag{O1}$$

O.1 Mutations emerging in resistant cells

We start by analysing S_k^R, which is the sum over all mutations arising in resistant clones $i = 1, ..., K$. Given expected CSD $E[\Theta_\kappa]$, we have

$$E[S_k^R] = E\left[\sum_{i=1}^{K} S_k^{R,i}\right] = \sum_{\kappa=1}^{N_d} E[\Theta_\kappa]\, E[S_k(\kappa)], \tag{O2}$$

where $E[S_k(\kappa)]$ denotes the expected SFS of a resistant clone with size κ excluding mutations that were acquired prior to obtaining the resistance mutation.

Before treatment, clones grow with constant birth and death rate analogously to a homogeneous population at detection. Thus, the fixed-time SFS of a single clone with size κ is described by

$$\begin{aligned} E[S_k(\kappa)] &= 2m\kappa \int_0^{1-1/\kappa} \left(1 - \frac{d_{r,1}}{b_{r,1}} y\right)^{-1} (1-y) y^{k-1}\, dy \\ &\to 2m\kappa \sum_{l=0}^{\infty} \frac{\left(\frac{d_{r,1}}{b_{r,1}}\right)^l}{(k+l)(k+l+1)} \qquad \text{for } \kappa \to \infty \\ &\to 2m\kappa \frac{b_{r,1}}{b_{r,1} - d_{r,1}} \frac{1}{k(k+1)} \qquad \text{for } k \to \infty. \end{aligned} \tag{O3}$$

During treatment, the growth rates of the resistant clones will change and $E[S_k(\kappa)]$ is more appropriately described by homogeneous population with continued increase given by eqn. (F7) and (F8). In the case of full resistance such that $b_r := b_{r,1} = b_{r,2}$ and $d_r := d_{r,1} = d_{r,2}$, we can keep the expression in eqn. (O3) for the treatment phase such that

$$E[S_k(\kappa)] \to 2m\kappa \sum_{l=0}^{\infty} \frac{\left(\frac{d_r}{b_r}\right)^l}{(k+l)(k+l+1)} \qquad \text{for } \kappa \to \infty, \tag{O4}$$

which once inserted into eqn. (O2) gives us the SFS of mutations that emerged in resistant cells. In particular, we then have

$$\begin{aligned} E[S_k^R] &= \sum_{i=1}^{K} E[S_k^{(R,i)}] \\ &\approx \sum_{i=1}^{K} 2m\kappa_i \sum_{l=0}^{\infty} \frac{\left(\frac{d_r}{b_r}\right)^l}{(k+l)(k+l+1)} \\ &= 2mN_r \sum_{l=0}^{\infty} \frac{\left(\frac{d_r}{b_r}\right)^l}{(k+l)(k+l+1)}, \end{aligned} \tag{O5}$$

where N_r is the number of resistant cells. If treatment is applied long enough, there will be no more sensitive cells and we have the total population given by the resistant cells $N_f = N_r$ giving us the neutral tail known from homogeneous populations.

O.2 Mutations emerging in sensitive cells

Obtaining the SFS of mutations that emerged in sensitive cells is more complicated. Neglecting the effect of resistance mutations on the growth of sensitive cells, the SFS of the sensitive cell population can be computed analogous to the homogeneous decreasing cell population whose expected SFS is described by eqn. (F1) and (F3). However, there are also mutations that emerged in sensitive cells, and are inherited to resistant cells.

For simplicity, we restrict ourselves on case that all sensitive cells went extinct. Then, mutations that emerged in sensitive cells must be clonal in at least one resistant clone, and we can write

$$S_k^S = \sum_{Y \in 2^X} C_Y 1_{\left\{\sum_{j\in Y} \kappa_j = k\right\}}. \tag{O6}$$

Here, the first sum goes over all possible combinations Y in the set of clone indices $X = 1, 2, ...K$ that is the power set 2^X, and C_Y is the number of mutations shared between the selected clones.

Using Theorem 3, we can connect the number of clonal mutations in a resistant clone i' to its arrival time $t_{\text{arrival}} \in [0, t_d + t_f]$. We have

$$E\left[\sum_{i' \in Y} C_Y\right] \approx \begin{cases} 2mb_{r,2}(t_d + t_f - t_{\text{arrival}}) & \text{if } t_{\text{arrival}} \in [t_d, t_f] \\ 2mb_{r,1}(t_d - t_{\text{arrival}}) + 2mb_{r,2}t_f & \text{if } t_{\text{arrival}} \in [0, t_d] \end{cases} \tag{O7}$$

To split this predictions into individual peaks C_Y in the SFS, one must study the relatedness between clone i' to other clones.

P Alternative models of mutation accumulation

Biologically, in the context of somatic evolution, it has been shown that mutation accumulation is a compound process with division-dependent and division-independent "background" mutations (Abascal *et al.* 2021). Division-induced mutations may act on one strand while background mutations act on both and mutations may fix in either daughter cell during division. Often, background mutations tend to dominate. These background mutations act clock-like and manifest as an apparent increase in the per-division mutation rate.

Theoretically, we can show that if mutation rates are sufficiently low, having a Poisson distribution for the mutation accumulation is equivalent to having a probability of resistance during division. Let us denote the mean of the Poisson distribution by m.

We show that in the limit of low mutation rates, namely $m \ll 1$, accumulation of 2 or more mutations within one division can be neglected. The Poisson distribution reads $p(k) = \frac{m^k e^{-m}}{k!}$ such that $p(k = 0) = e^{-m}$ and $p(k = 1) = me^{-m}$. We expand the exponential function using its power series, $e^{-m} = 1 - m + \frac{m^2}{2} + O(m^3)$. The probability to obtain more than 1 mutation is given by $p(k \geq 2) = 1 - p(k = 0) - p(k =$

1). Using the power series of the exponential function, this reads $p(k \geq 2) = \frac{m^2}{2} + O(m^3) = O(m^2)$. Thus, by neglecting the chance of obtaining 2 or more mutations, we neglect events in the order $O(m^2)$ that are negligible when $m \ll 1$. Furthermore, we have $p(k = 1) = me^{-m} = m\left(1 - m + O(m^2)\right) = m + O(m^2)$. In words, the expected number of mutations coincides with the probability of obtaining one mutation in linear order to m.

In the low mutation rate limit, it also makes no difference whether mutations are accumulated in both or only one daughter cell during division. Using a similar argumentation as above, the chance that both daughter cells obtain a mutation during division is of the order m^2 and is thus negligible. However, a correcting factor 2 must be added if mutations are accumulated on only in one daughter cell to obtain the same mutation rate per division.

If mutation rates are of magnitude 1 or higher per division, we expect differences in some but not all measures of gITH. In expectation, as shown by (Gunnarsson *et al.* 2021), the SFS remains unchanged as it only depends on the expected number of mutations acquired during a single division. The expected scMB distribution would change because the variance of the distribution depends on some of the underlying biology.

Literature cited

Abascal F, Harvey LM, Mitchell E, Lawson AR, Lensing SV, Ellis P, Russell AJ, Alcantara RE, Baez-Ortega A, Wang Y *et al.* 2021. Somatic mutation landscapes at single-molecule resolution. Nature. 593:405–410.

Antal T, Krapivsky P. 2011. Exact solution of a two-type branching process: models of tumor progression. Journal of Statistical Mechanics: Theory and Experiment. 2011:P08018.

Bailey NT. 1991. *The elements of stochastic processes with applications to the natural sciences*. volume 25. John Wiley & Sons.

Butler RW. 2007. *Saddlepoint approximations with applications*. volume 22. Cambridge University Press.

Cheek D, Antal T. 2018. Mutation frequencies in a birth–death branching process. The Annals of Applied Probability. 28:3922–3947.

Cheek D, Johnston SG. 2023. Ancestral reproductive bias in branching processes. Journal of Mathematical Biology. 86:70.

Clauset A, Shalizi CR, Newman ME. 2009. Power-law distributions in empirical data. SIAM review. 51:661–703.

Consul PC, Jain GC. 1973. A generalization of the poisson distribution. Technometrics. 15:791–799.

Daniels HE. 1954. Saddlepoint approximations in statistics. The Annals of Mathematical Statistics. pp. 631–650.

David HA, Nagaraja HN. 2004. *Order statistics*. John Wiley & Sons.

Davison AC, Hautphenne S, Kraus A. 2021. Parameter estimation for discretely observed linear birth-and-death processes. Biometrics. 77:186–196.

Durrett R. 2008. *Probability models for DNA sequence evolution*. volume 2. Springer.

Durrett R. 2015. *Branching process models of cancer*. Springer.

Gunnarsson EB, Leder K, Foo J. 2021. Exact site frequency spectra of neutrally evolving tumors: A transition between power laws reveals a signature of cell viability. Theoretical Population Biology. 142:67–90.

Kessler DA, Levine H. 2013. Large population solution of the stochastic luria–delbrück evolution model. Proceedings of the National Academy of Sciences. 110:11682–11687.

Kessler DA, Levine H. 2015. Scaling solution in the large population limit of the general asymmetric

stochastic luria–delbrück evolution process. Journal of statistical physics. 158:783–805.
Moeller ME, Père NVM, Werner B, Huang W. 2024. Measures of genetic diversification in somatic tissues at bulk and single-cell resolution. eLife. 12:RP89780.
Nicholson MD, Cheek D, Antal T. 2023. Sequential mutations in exponentially growing populations. PLOS Computational Biology. 19:e1011289.
Ohtsuki H, Innan H. 2017. Forward and backward evolutionary processes and allele frequency spectrum in a cancer cell population. Theoretical Population Biology. 117:43–50.
Tavaré S. 2018. The linear birth–death process: an inferential retrospective. Advances in Applied Probability. 50:253–269.
Williams MJ, Werner B, Heide T, Curtis C, Barnes CP, Sottoriva A, Graham TA. 2018. Quantification of subclonal selection in cancer from bulk sequencing data. Nature genetics. 50:895–903.

Supplementary figures

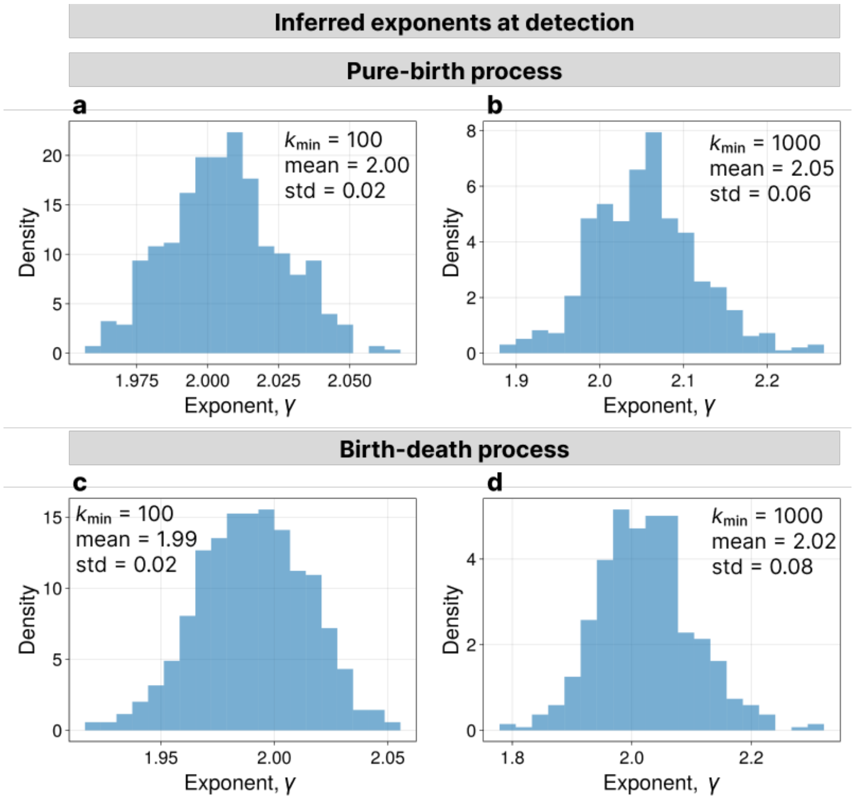

Supplementary Fig. S1: Fitted exponents to the SFS of simulated populations at detection using eqn. (39). a) Parameters: $b_1 = 1.0$, $d_1 = 1.0$, $m = 2.0$, $N_d = 10^5$ and $k_{\text{min}} = 100$. b) Parameters: $b_1 = 1.0$, $d_1 = 1.0$, $m = 2.0$, $N_d = 10^5$ and $k_{\text{min}} = 1000$. c) Parameters: $b_1 = 1.0$, $d_1 = 0.7$, $m = 2.0$, $N_d = 10^5$ and $k_{\text{min}} = 100$. d) Parameters: $b_1 = 1.0$, $d_1 = 0.7$, $m = 2.0$, $N_d = 10^5$ and $k_{\text{min}} = 1000$. Simulations were repeated 500 times.

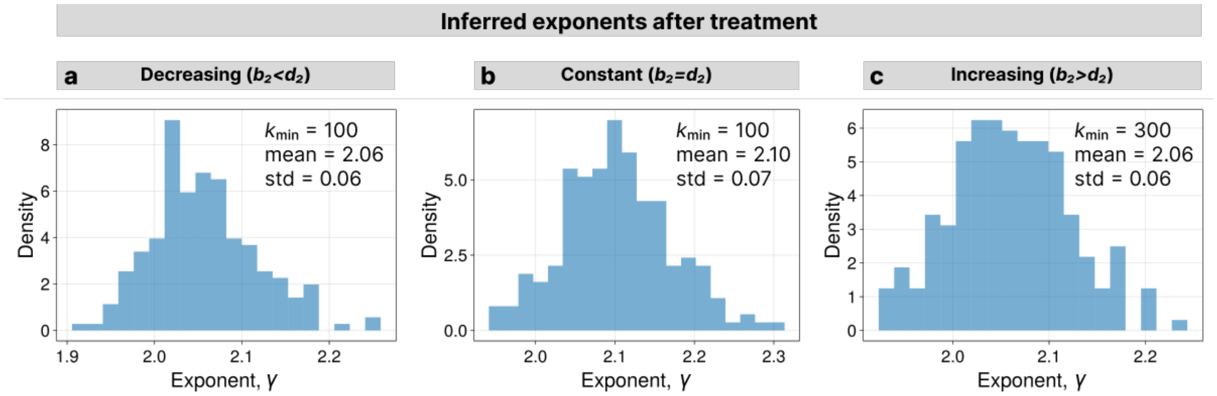

Supplementary Fig. S2: Fitted exponents to the SFS in of simulated homogeneous populations after treatment using eqn. (39). a) For decreasing population. Parameters before treatment: $b_1 = 1.0$, $d_1 = 0.0$, $m = 2.0$ and $N_d = 10^5$. Parameters during treatment: $b_2 = 1.0$, $d_2 = 3.0$, $m = 2.0$ and $N_f = 10^4$. Parameter for inference: $k_{\min} = 100$. b) For constant population. Parameters before treatment: $b_1 = 1.0$, $d_1 = 0.0$, $m = 2.0$ and $N_d = 10^5$. Parameters during treatment: $b_2 = d_2 = 1.0$, $m = 2.0$ and $t_f = 8.0$. Parameter for inference: $k_{\min} = 100$. c) For increasing population. Parameters before treatment: $b_1 = 1.0$, $d_1 = 0.0$, $m = 2.0$ and $N_d = 10^4$. Parameters during treatment: $b_2 = 1.0$, $d_2 = 0.6$, $m = 2.0$ and $N_f = 3.0 \times 10^4$. Simulations were repeated 200 times for each treatment scenario. Parameter for inference: $k_{\min} = 300$. Simulations were repeated 200 times for each treatment scenario.

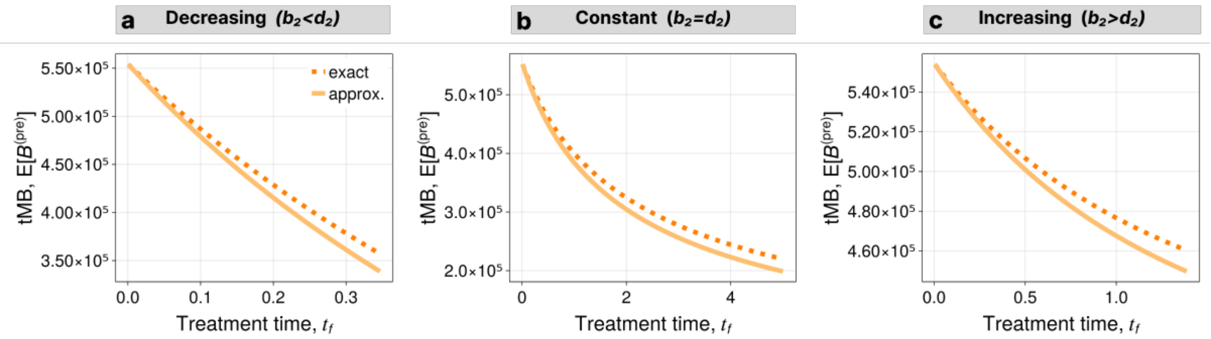

Supplementary Fig. S3: Comparison of approximate and exact expressions for the expected tMB of preexisting mutations after homogeneous treatment response. Exact tMB is given by eqn. (J2) and approximate tMB is given by eqn. (22). Parameters before treatment: $b_1 = 1.0$, $d_1 = 0.5$, $m = 2.0$ and $N_d = 10^5$. Parameters during treatment for decreasing population: $b_2 = 1.0$, $d_2 = 3.0$, $m = 2.0$ and t_f varied. Parameters during treatment for constant population: $b_2 = d_2 = 1.0$, $m = 2.0$ and t_f varied. Parameters during treatment for increasing population: $b_2 = 1.0$, $d_2 = 0.5$, $m = 2.0$ and t_f varied.

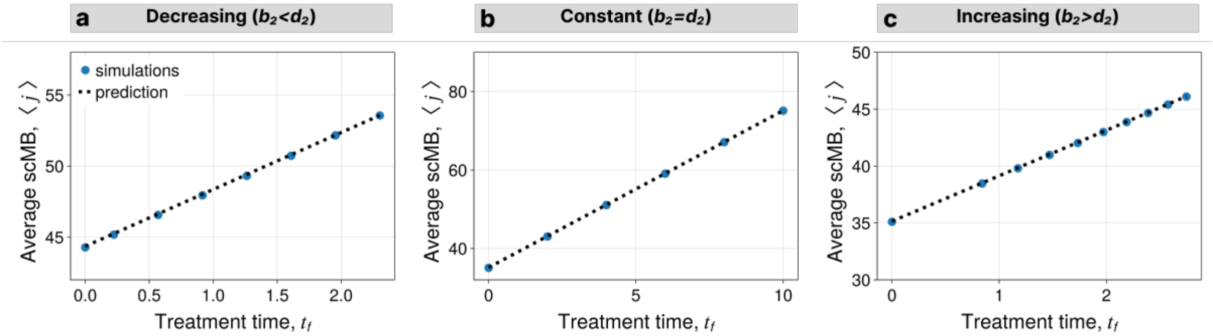

Supplementary Fig. S4: Average scMB after treatment for homogeneous population. a) For decreasing population. Parameters before treatment: $b_1 = 1.0$, $d_1 = 0.0$, $m = 2.0$ and $N_d = 10^5$. Parameters during treatment: $b_2 = 1.0$, $d_2 = 3.0$, $m = 2.0$ and N_f is varied. b) For constant population. Parameters before treatment: $b_1 = 1.0$, $d_1 = 0.0$, $m = 2.0$ and $N_d = 10^5$. Parameters during treatment: $b_2 = d_2 = 1.0$, $m = 2.0$ and t_f is varied. c) For increasing population. Parameters before treatment: $b_1 = 1.0$, $d_1 = 0.0$, $m = 2.0$ and $N_d = 10^4$. Parameters during treatment: $b_2 = 1.0$, $d_2 = 0.6$, $m = 2.0$ and N_f is varied. Simulations were repeated 200 times.

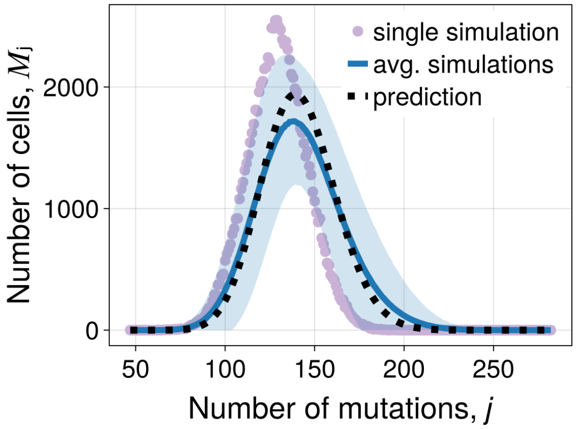

Supplementary Fig. S5: Single cell mutational burden distribution at detection for birth-death process. Parameters: $b_1 = 1.0$, $d_1 = 0.7$, $m = 2.0$ and $N_d = 10^5$. Simulations were repeated 500 times.

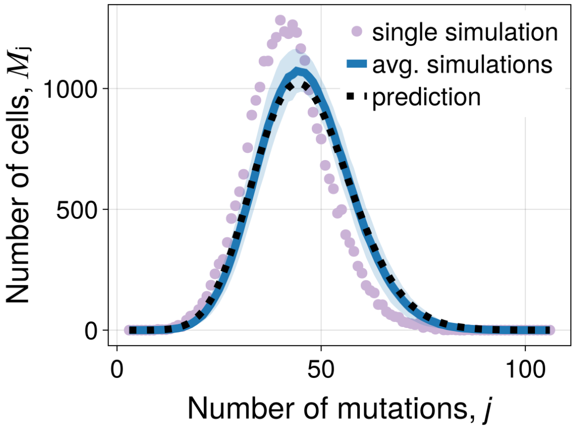

Supplementary Fig. S6: Single cell mutational burden distribution for continued increasing population. Parameters before treatment: $b_1 = 1.0$, $d_1 = 0.0$, $m = 2.0$ and $N_d = 10^4$. Parameters during treatment: $b_2 = 1.0$, $d_2 = 0.6$, $m = 2.0$ and $N_f = 3.0 \times 10^4$. Simulations were repeated 200 times.

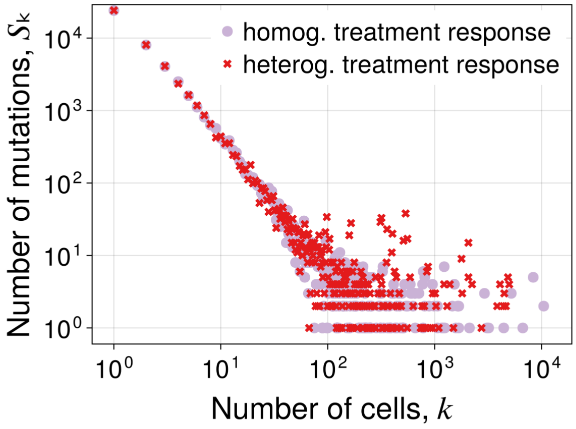

Supplementary Fig. S7: SFS of homogeneous vs heterogeneous population. For homogeneous population, we use parameters $b_1 = 1.0$, $d_1 = 0.0$, $m = 2.0$, $N_d = 1.2 \times 10^4$. For the heterogeneous population, we use parameters before treatment $b_{s_1} = b_{r_1} = 1.0$, $d_{s_1} = d_{r_1} = 0.0$, $m = 2.0$, $\nu = 10^{-3}$, $N_d = 10^4$ and during treatment $b_{s_2} = b_{r_2} = 1.0$, $d_{s_2} = 3.0$, $d_{r_2} = 0.0$, $m = 2.0$, $\nu = 10^{-3}$, $N_f = 1.2 \times 10^4$. The shown SFS is the result of a single realisation at the end of each scenario.

Supplementary tables

Figure	b_1	d_1	N_d	b_2	d_2	N_f	t_f	m	realisations
2a	1.0	0.7	10^5	–	–	–	–	2.0	500
2b	1.0	0.0	10^5	–	–	–	–	2.0	500
2c	1.0	0.0	10^5	–	–	–	–	2.0	500
2d	1.0	0.0	10^5	1.0	3.0	10^4	–	2.0	200
2e	1.0	0.0	10^5	1.0	1.0	–	8.0	2.0	200
2f	1.0	0.0	10^4	1.0	0.6	3×10^4	–	2.0	200

Supplementary Table S1: Parameters for Fig. 2.

Figure	b_1	d_1	N_d	t_d	b_2	d_2	N_f	t_f	m	realisations
3a	1.0	0.0	10^5	–	–	–	–	–	2.0	500
3b	1.0	0.7	10^5	–	–	–	–	–	2.0	500
3c	1.0	0.0	–	9.21	–	–	–	–	2.0	500
3d	1.0	0.0	10^5	–	1.0	3.0	varied	–	2.0	200
3e	1.0	0.0	10^5	–	1.0	1.0	–	varied	2.0	200
3f	1.0	0.0	10^4	–	1.0	0.6	varied	–	2.0	200

Supplementary Table S2: Parameters for Fig. 3.

Figure	b_1	d_1	N_d	b_2	d_2	N_f	t_f	m	realisations
4a	1.0	0.0	10^5	–	–	–	–	2.0	500
4b	1.0	0.0	10^5	1.0	3.0	10^4	–	2.0	200
4c	1.0	0.0	10^5	1.0	1.0	–	8.0	2.0	200

Supplementary Table S3: Parameters for Fig. 4.

Figure	b_1	d_1	N_d	t_d	$b_{s,2}$	$d_{s,2}$	$b_{r,2}$	$d_{r,2}$	N_f	m	ν	realisations
5a	0.14	0.13	–	2555	–	–	–	–	–	–	10^{-7}	10^6
5b	1.0	0.5	10^5	–	–	–	–	–	–	–	10^{-3}	500
5c	0.14	0.13	varied	–	–	–	–	–	–	–	10^{-7}	500
5d	1.0	0.0	10^4	–	1.0	3.0	1.0	0.0	2.0×10^4	2.0	10^{-3}	200
5e	1.0	0.0	10^4	–	1.0	3.0	1.0	0.0	2.0×10^4	2.0	10^{-3}	200
5f	1.0	0.0	10^4	–	1.0	1.5	0.7	0.0	1.2×10^4	2.0	10^{-3}	1

Supplementary Table S4: Parameters for Fig. 5. We set $b_{s,1} = b_{r,1} = b_1$ and $d_{s,1} = d_{r,1} = d_1$.

Growth rates	$b_s = 1.0$<br>$d_s = 0.0$<br>$b_r = 1.0$<br>$d_r = 0.0$	$b_s = 1.0$<br>$d_s = 0.5$<br>$b_r = 1.0$<br>$d_r = 0.5$	$b_s = 1.0$<br>$d_s = 0.0$<br>$b_r = 1.0$<br>$d_r = 0.2$	$b_s = 1.0$<br>$d_s = 0.0$<br>$b_r = 1.0$<br>$d_r = 0.5$	$b_s = 1.0$<br>$d_s = 0.5$<br>$b_r = 1.0$<br>$d_r = 0.6$
$f_{\{\tilde{\kappa}_2 > \tilde{\kappa}_1\}}$	32%	32%	33%	40%	36%
$E[\tilde{\kappa}_1 - \tilde{\kappa}_2]$	279	568	122	11	228
$std[\tilde{\kappa}_1 - \tilde{\kappa}_2]$	853	1348	403	58	815
$Med[\tilde{\kappa}_1 - \tilde{\kappa}_2]$	91	128	30	4	39
$Med[\tilde{\kappa}_1]$	174	328	63	15	115
$Med[\tilde{\kappa}_2]$	76	145	30	11	61
$\frac{Med[\tilde{\kappa}_1]}{Med[\tilde{\kappa}_2]}$	2.30	2.25	2.10	1.36	1.86

Supplementary Table S5: Comparison between the sizes of the first and second arriving subclone in simulated tumours at detection for different parameter settings. Specifically, we denote size of the first arriving clone by $\tilde{\kappa}_1$ and the size of the second clone by $\tilde{\kappa}_2$. We then compute the fraction of simulations in which the second arriving clone is larger than the first, $f_{\{\tilde{\kappa}_2 > \tilde{\kappa}_1\}}$, the expected size difference $E[\tilde{\kappa}_1 - \tilde{\kappa}_2]$, the standard deviation in the size difference $std[\tilde{\kappa}_1 - \tilde{\kappa}_2]$, the median size difference, $Med[\tilde{\kappa}_1 - \tilde{\kappa}_2]$, the individual median sizes, $Med[\tilde{\kappa}_1]$ and $Med[\tilde{\kappa}_2]$ as well as the ratio of the latter two $\frac{Med[\tilde{\kappa}_1]}{Med[\tilde{\kappa}_2]}$. We fix $N_d = 10^4$ and $\nu = 10^{-2}$. Every parameter set was simulated 500 times.
